# Gestational Diabetes, the Human Milk Metabolome, and Infant Growth and Adiposity

**DOI:** 10.1001/jamanetworkopen.2024.50467

**Published:** 2024-12-12

**Authors:** Emily M. Nagel, Armando Peña, Jonathan M. Dreyfuss, Eric F. Lock, Kelsey E. Johnson, Chang Lu, David A. Fields, Ellen W. Demerath, Elvira Isganaitis

**Affiliations:** 1 School of Public Health, University of Minnesota, Twin Cities; 2 Department of Pediatrics, University of Minnesota, Minneapolis; 3 Department of Health and Wellness Design, School of Public Health, Indiana University Bloomington, Bloomington; 4 Joslin Diabetes Center, Harvard Medical School, Boston, Massachusetts; 5 Division of Biostatistics and Health Data Science, School of Public Health, University of Minnesota-Twin Cities; 6 Department of Genetics, Cell Biology, and Development, University of Minnesota-Twin Cities; 7 Division of Endocrinology, Boston Children’s Hospital, Harvard Medical School, Boston, Massachusetts; 8 Department of Pediatrics, University of Oklahoma College of Medicine

## Abstract

**Importance:**

Gestational diabetes (GD) is linked to health risks for the birthing parent and infant. The outcomes of GD on human milk composition are mostly unknown.

**Objective:**

To determine associations between GD, the human milk metabolome, and infant growth and body composition.

**Design, Setting, and Participants:**

Cohort study using data from the Mothers and Infants Linked for Healthy Growth and the Maternal Milk, Metabolism, and the Microbiome studies at the University of Oklahoma and University of Minnesota, large prospective US cohorts with a high proportion of exclusive breastfeeding. Participants were mother-infant dyads recruited between October 2014 and August 2019 who planned to exclusively breastfeed for 3 or more months. Data were analyzed from July 2022 to August 2024.

**Exposure:**

GD diagnosed via oral glucose tolerance test.

**Main Outcomes and Measures:**

The milk metabolome was assessed by untargeted liquid chromatography–gas chromatography–mass spectrometry at 1 month post partum. Infant growth (weight for length *z* score, length for age *z* score, and rapid weight gain) and body composition (percentage body fat and fat-free mass index) from 0 to 6 months were assessed. Linear regression analyses tested associations between GD and milk metabolites, with adjustment for covariates and potential confounders.

**Results:**

Among 348 dyads (53 with GD), 27 (51%) of the GD-exposed infants were female and 157 (53%) of nonexposed infants were male; 10 (19%) were Asian, 2 (4%) were Black or African American, and 37 (70%) were White. The mean (SD) age was higher in the GD group (with GD, 34.0 [4.3] years; without GD, 30.7 [4.1] years). In adjusted models, GD was associated with differential levels of 9 metabolites of 458 tested (FDR<0.05); 3 were higher (2-hydroxybutyric acid, 3-methylphenylacetic acid, and pregnanolone sulfate) and 6 were lower in women with GD (4-cresyl sulfate, cresol, glycine, P-cresol sulfate, phenylacetic acid, and stearoylcarnitine). Phenylacetic acid was associated with length for age *z* score (β = 0.27; SE, 0.13; 95% CI, 0.02 to 0.16), 2-hydroxybutryic acid with percentage body fat (β = −1.50; SE, 0.66; 95% CI, −2.79 to −4.82), and stearoylcarnitine with greater odds of rapid weight gain (odds ratio, 1.66; 95% CI, 1.23 to 2.25). GD was associated with greater length for age *z* scores (β = 0.48; SE, 0.22; 95% CI, 0.04 to 0.91).

**Conclusions and Relevance:**

In this observational cohort study, GD was associated with altered concentrations of several human milk metabolites. The associations between these metabolites and infant growth suggest that milk compositional differences in mothers with GD may beneficially moderate the growth and body composition of their infants.

## Key Points

**Question:**

Does the human milk metabolome differ by gestational diabetes (GD) status in ways that are associated with infant growth and body composition?

**Findings:**

In this cohort study of 348 predominantly breastfeeding mother-infant dyads, GD was associated with differences in several human milk metabolite abundances that were in turn associated with faster linear growth and lower adiposity.

**Meaning:**

The associations identified in this study between milk metabolomics variation and infant growth suggest that milk compositional differences in mothers with GD may beneficially moderate the growth and body composition of their infants.

## Teaser

This cohort study evaluates associations between gestational diabetes, the human milk metabolome, and infant growth and body composition.

## Introduction

The prevalence of gestational diabetes (GD) has risen sharply in recent years, now affecting over 8% of pregnancies in the US.^1^ This is concerning because GD is linked to suboptimal health for both the infant and the birthing parent (eg, increased risk of diabetes, cardiovascular disease, and obesity).^2^ However, there is evidence that breastfeeding can moderate these relationships by improving maternal postpartum glucose metabolism and insulin sensitivity.^3^ Human milk composition has also been hypothesized to mediate the beneficial effects of breastfeeding on offspring adiposity.^4^ Some studies have reported lower infant weight gain and/or childhood obesity in GD-exposed offspring that were breastfed.^4,5^ Conversely, other studies have suggested that the protective effects of breastfeeding on offspring adiposity are attenuated in mothers with GD, and follow-up of GD-exposed infants showed that ingesting higher amounts of maternal milk (vs non-GD donor milk) led to higher body mass index (BMI) and insulin resistance at 2 years.^6,7,8^ Thus, whether GD leads to meaningful alterations in human milk composition that influence childhood growth and adiposity is unclear.

Several milk components, including the hormones ghrelin, insulin, and adiponectin, and various lipids and human milk oligosaccharides, have been associated with GD status, but the relationship between GD-associated milk components and infant growth remains unclear.^9,10,11,12,13,14^ Metabolomic analysis, with its assessment of hundreds of low molecular weight components active in numerous metabolic pathways, provides a comprehensive approach to testing the hypothesis that maternal metabolic health is associated with human milk in ways that may either program or moderate infant adiposity and health. We are aware of only 2 previous studies examining the relationship between GD and the human milk metabolome,^15,16^ 1 of which also explored associations with infant growth.^16^ However, no data were available on maternal diet or infant body composition.

In a large contemporary US-based pregnancy cohort of mothers and infants with a high proportion of exclusive breastfeeding, we analyzed differences in the human milk metabolome by GD status. For metabolites that differed by GD, we tested associations with infant growth and body composition in the first 6 months. We hypothesized that GD would be associated with differences in the human milk metabolome and that these differential metabolites would be associated with infant growth and body composition.

## Methods

The institutional review boards at the University of Minnesota, Health Partners Institute, and the University of Oklahoma Health Sciences Center approved all Mothers and Infants Linked for Healthy Growth (MILK) Study (NCT03301753) and Maternal Milk, Metabolism, and the Microbiome (4M) Study (NCT03522597) protocols. Participants provided written informed consent and were compensated for each study visit. This study followed Strengthening the Reporting of Observational Studies in Epidemiology (STROBE) reporting guidelines for cohort studies.

### Study Population

Data from mother-infant dyads enrolled in the ongoing, prospective MILK study and the 4M study between October 2014 and August 2019 were included in these analyses. Inclusion criteria were mothers aged 21 to 45 years at delivery, prepregnancy BMI 18.5 to 45.0 (calculated as weight in kilograms divided by height in meters squared), healthy singleton pregnancy, full-term infant, and intention and social support to breastfeed for 3 or more months. Participants were excluded if they consumed tobacco or more than 1 alcoholic drink weekly during pregnancy and/or lactation, were unable to speak or understand English, or if their infants had a congenital illness known to affect feeding and/or growth (eg, genetic abnormalities [eg, trisomy 21 or cystic fibrosis], inborn errors of metabolism [eg, phenylketonuria or galactosemia], or structural or neurological abnormalities that could affect infant feeding [eg, congenital heart conditions, cerebral palsy, cleft lip, and so forth]). Participants were enrolled in the third trimester of pregnancy after GD status was ascertained. By design, all participants were exclusively breastfeeding at 1 month post partum, 93% at 3 months, and 75% at 6 months. Of the enrolled 415 dyads that had completed the study at the time of metabolomics analysis, all women with GD, all women from minoritized racial and ethnic groups without GD, and a random selection of the remaining women without GD in the cohort were included (participants were randomly selected using PROC SURVEYSELECT in SAS). Funding was available to analyze 350 milk samples, and 348 of these were included in the final dataset (2 dyads were excluded due to nonexclusive breastfeeding status at 1 month post partum) (eTable 1 in Supplement 1).

### Maternal Characteristics

Participant characteristics (Table) were collected from electronic medical records, including GD diagnosis, parity (0 or ≥1), delivery mode (vaginal or cesarean section), prepregnancy BMI, age at expected date of delivery, gestational weight gain, and postpartum weight loss. Maternal race and ethnicity (American Indian or Alaska Native, Asian, Black or African American, Hispanic/Latino, non-Hispanic/Latino, White, more than 1 race, unknown, and other races and ethnicities [no further information available; participants chose “other”]), household income (<$60 000, $60 000-$90 000, or >$90 000), and education level (high school/GED/associate’s degree, bachelor’s degree, or graduate degree) were obtained by maternal self-report. Race and ethnicity were assessed to ensure diversity and representation in the study cohort and because National Institutes of Health–funded projects are required to report race and ethnicity data for study participants. Maternal diet quality scores were calculated from the National Cancer Institute’s Diet History Questionnaire II (completed at the 1-month post partum visit) using the Healthy Eating Index 2015.^17,18^

**Table.** Participant Characteristics

Characteristic	Participants, No. (%)	
	GD (n = 53)	Non-GD (n = 295)
Study site		
Minnesota	50 (94)	177 (60)
Oklahoma	3 (6)	118 (40)
Maternal age, mean (SD), y	34.0 (4.3)	30.7 (4.1)
Maternal race		
American Indian or Alaska Native	1 (2)	3 (1)
Asian	10 (19)	8 (3)
Black or African American	2 (4)	16 (5)
White	37 (70)	242 (83)
More than 1 race	1 (2)	9 (3)
Other races and ethnicities^a^	1 (2)	12 (4)
Unknown	1 (2)	3 (1)
Maternal ethnicity		
Hispanic/Latino	2 (4)	9 (3)
Not Hispanic/Latino	51 (96)	282 (96)
Unknown	0	2 (1)
Maternal education		
High school/GED/associate’s degree	12 (23)	72 (25)
Bachelor’s degree	21 (40)	110 (39)
Graduate degree	20 (37)	101 (36)
Annual income status, US $		
<60 000	12 (23)	92 (33)
60 000-90 000	6 (11)	69 (24)
>90 000	35 (66)	122 (43)
Parity		
0	19 (37)	109 (38)
≥1	32 (63)	179 (62)
Prepregnancy body mass index, median (IQR)^b^	28.8 (23.4-34.7)	26.0 (22.4-30.1)
<25	18 (34)	118 (40)
25-29	10 (19)	95 (32)
≥30	25 (47)	80 (27)
Oral glucose challenge test, mean (SD), mg/dL	159.0 (19.8)	106.7 (18.2)
Gestational weight gain, mean (SD), kg	9.52 (4.76)	12.10 (6.75)
Postpartum weight loss at 1 mo, mean (SD), kg	9.73 (2.57)	8.54 (4.76)
Mode of delivery		
Vaginal	31 (58)	218 (75)
Cesarean section	22 (42)	71 (25)
Exclusive breastfeeding		
3 mo	41 (91)	263 (93)
6 mo	32 (78)	205 (75)
Diet quality score, mean (SD)	62.5 (10.9)	65.3 (8.8)
Infant gestational age at birth, median (IQR) wk	39.0 (38.0-39.3)	39.7 (39.0-40.4)
Birth anthropometrics, mean (SD)		
Weight, kg	3.31 (0.42)	3.53 (0.44)
Weight for age *z* score	0.016 (0.83)	0.46 (0.88)
Length, cm	51.1 (2.3)	51.7 (2.2)
Length for age *z* score	0.83 (1.19)	1.15 (1.18)
Weight for length *z* score	−0.91 (1.43)	−0.68 (1.39)
Rapid infant weight gain from 0-6 mo^c^	4 (10)	39 (15)
Infant sex		
Male	26 (49)	157 (53)
Female	27 (51)	138 (47)
Infant fat mass, mean (SD), kg		
1 mo^d^	0.77 (0.32)	0.80 (0.29)
3 mo^d^	1.48 (0.47)	1.48 (0.41)
6 mo^e^	2.77 (0.68)	2.77 (0.50)
Infant fat-free mass, mean (SD), kg		
1 mo^d^	3.54 (0.43)	3.75 (0.44)
3 mo^d^	4.49 (0.49)	4.69 (0.52)
6 mo^e^	5.47 (0.63)	5.31 (0.59)
Infant percentage body fat, mean (SD), %		
1 mo^d^	17.5 (5.3)	17.2 (4.9)
3 mo^d^	24.5 (5.3)	23.7 (4.8)
6 mo^e^	33.3 (4.9)	34.0 (3.4)

Abbreviation: GD, gestational diabetes.

SI conversion factor: To convert glucose to mmol/L, multiply by 0.0555.

^a^ Study participants self-identified race and could select other if they did not identify with any of the provided options; there is no further information regarding the categories included in other.

^b^ Body mass index is calculated as weight in kilograms divided by height in meters squared.

^c^ Rapid weight gain: change in weight for age z score from birth to 6 months greater than 0.67.

^d^ Obtained via air displacement plethysmography.

^e^ Obtained via dual energy x-ray absorptiometry.

GD status was determined using electronic medical records data. A 1-hour blood glucose concentration was obtained after a 50-g oral glucose challenge test (OGCT) administered between 26 and 28 weeks of gestation to screen for GD. Women with OGCT levels higher than 130 mg/dL received a confirmatory 3-hour 100-g oral glucose tolerance test. GD was diagnosed if 2 or more of the following 4 glucose levels were met: 95 mg/dL and higher (fasting), 180 mg/dL and higher (1 hour), 155 mg/dL and higher (2 hours), and 140 mg/dL and higher (3 hours) (to convert glucose to mmol/L, multiply by 0.0555).^19^ A total of 26 participants with GD (49%) were treated via insulin, 24 (45%) via dietary modifications, 2 (4%) via oral medication, and 1 (2%) via both insulin and oral medication. Patients without GD randomly selected for the present study were similar to participants without GD not selected for inclusion (eTable 2 in Supplement 1).

### Infant Data

Infant gestational age at birth, birth weight, sex, and age at study visit were obtained from electronic medical records. All infants were exclusively breastfeeding at 1 month post partum and were evaluated for breastfeeding status at 3- and 6-month study visits by maternal self-report. Infants were considered exclusively breastfeeding if they received less than 24 oz of formula since birth or their last study visit and only human milk, water, and vitamins for 2 weeks before the study visit. Anthropometric and body composition measurements were obtained at 1-, 3-, and 6-month study visits as described previously.^20^ All infant anthropometric data underwent quality control to exclude implausible values. World Health Organization growth charts were used to obtain *z* scores for anthropometric measures (weight for age *z* score [WAZ], length for age *z* score [LAZ], and weight for length *z* score [WLZ]). At 1 and 3 months, air displacement plethysmography (ADP) was used to assess infant body composition (fat mass, fat-free mass, [FFM], and percentage body fat [%BF]) via the Pea Pod device (Cosmed Ltd).^21^ At 6 months, dual energy x-ray absorptiometry (Lunar iDXAv11-30.062 scanner; analysis via enCore 2007 software, GE HealthCare) was used to assess body composition. Because *z* scores are only available for ADP-generated body composition measures, raw body composition values were used in analyses for consistency.

### Human Milk Collection

At 1 month (±5 days), participants attended study visits between 8:00 and 10:00 am. Mothers were instructed to breastfeed their infant ad libitum from 1 or both breasts per usual practice. Two hours after feeding, each mother provided a sample from a single complete right breast expression (until milk flow stopped) using a hospital-grade electric breast pump (Medela Symphony, Medela, Inc) to ensure collection of fore-, mid-, and hind-milk within each sample.^22^ The volume and weight of the single breast expression were recorded before the individual samples were gently mixed, aliquoted, and stored at −80 °C within 20 minutes of collection.

### Metabolomic Analyses

Analyses to quantify relative abundances of human milk metabolites were performed using untargeted liquid chromatography–gas chromatography–mass spectrometry (BPGbio) (eMethods in Supplement 1).^23^ Samples were run in 10 batches (nonstop), with each batch including 10 pooled quality control (QC) and 40 external QC standards (mean [range] % coefficient of variation [CV], 33% [3%-221%]) (eTable 1 in Supplement 1). The ComBat method was used to adjust for batch effects.^24^ Metabolites with missing values in more than 20% of samples were removed from further analysis. Imputation of missing values was performed using routine replacement by limits of detection (1/5 the minimum positive value of each variable). Metabolomics data were normalized across samples by median centering and were log transformed (base 10) before analysis.

### Statistical Analyses

#### Participant Characteristics

Participant characteristics were evaluated as raw mean and SD (continuous variables) or frequency and percentage (categorical variables). Continuous variables were compared using unpaired *t* tests (normally distributed variables) or Wilcoxon rank sum tests (nonnormally distributed), while categorical variables were compared using χ^2^ tests.

#### Primary Analysis: Maternal GD and Milk Metabolite Abundances

Metabolite profiles were visualized using principal component analysis plots. Univariable and multiple linear regression analyses were used to test the associations between maternal GD (exposure) and 1-month human milk metabolite abundances (outcome). Models (after checking for the presence of multicollinearity) were adjusted for known covariates and potential confounders from the literature, including study center (Minnesota or Oklahoma) and maternal age, prepregnancy BMI, household income, education level, parity, and infant age at visit and infant sex (eFigure 1 in Supplement 1). Data missingness ranged from less than 1% to 11% for individual variables (eTable 3 in Supplement 1). Participants with complete data were included in adjusted models. To complement our analysis of GD and milk metabolites, we also tested continuous associations between maternal OGCT and metabolite abundances. For all analyses, Benjamini-Hochberg procedure was used to adjust *P* values for false discovery rate (FDR), with an FDR-corrected *P* < 0.05 considered significant.^25^ Among metabolites significantly associated with maternal GD (*P* < .05), pathway enrichment analyses were conducted, using all 458 milk metabolites detected in the metabolomic analysis as the reference metabolome (MetaboAnalyst 5.0^26^).

#### Secondary Analysis: Milk Metabolite Abundances and Infant Growth and Body Composition

Linear regression models were constructed to test the associations between the metabolites significantly associated with GD and infant growth and body composition, including change in WLZ and LAZ from 0 to 6 months, change in %BF from 1 to 3 months, %BF at 6 months, and FFM index at 6 months (FFM divided by length squared). Logistic regression analyses were used to test the association between milk metabolites associated with GD and rapid weight gain (RWG) from 0 to 6 months (change in WAZ > 0.67).^27^ Models were adjusted for covariates and potential confounders, including GD, study center, parity, maternal age, delivery category, education level, income category, prepregnancy BMI, and infant gestational age at birth (eFigure 2 in Supplement 1). Body composition models were further adjusted for infant age at study visit and sex. Two-sided *P* values less than .05 were considered statistically significant. R Studio version 2023.06.2 (R Project for Statistical Analysis) and SAS Enterprise Guide version 8.3 (SAS Institute) were used to conduct statistical analyses. Data were analyzed from July 2022 to August 2024.

## Results

### Participant Characteristics

Among 348 dyads (53 with GD), 27 (51%) of the GD-exposed infants were female and 157 (53%) of nonexposed infants were male; 10 (19%) were Asian, 2 (4%) were Black or African American, and 37 (70%) were White. Most mothers had received a college or graduate education (41 [77%] with GD and 211 [75%] without). The mean (SD) age was higher in the GD group (with GD, 34.0 [4.3] years; without GD, 30.7 [4.1] years; [data]; *P* < .001) whereas parity was similar. A higher percentage of study participants with GD delivered via cesarean section (22 [42%] with GD and 71 [25%] without; *P* = .02) (eTable 4 in Supplement 1). Prepregnancy mean (SD) BMI was higher in the GD group (GD, 29.4 [6.6]; non-GD, 26.9 [5.8]; *P* = .01), while exclusive breastfeeding proportions were similar between GD and non-GD groups at 1, 3, and 6 months post partum. Maternal diet quality scores were not significantly different between groups (eTable 4 in Supplement 1).

Infant sex (with GD, 27 [51%] male; without GD, 157 [53%] male) was similar between groups, while mean (SD) gestational age at birth (GD, 38.6 [1.2] weeks; non-GD, 39.6 [1.2] weeks; *P* = <.001) and birth weight (GD, 3.31 [0.42] kg; non-GD, 3.53 [0.44] kg; *P* = .001) were slightly lower in the GD group. The percentage of infants with RWG from 0 to 6 months was lower in the GD group (4 infants [10%] vs 39 infants without GD [15%]).

### Associations of Maternal GD Status and OGCT With Metabolite Abundances

**Figure 1.**
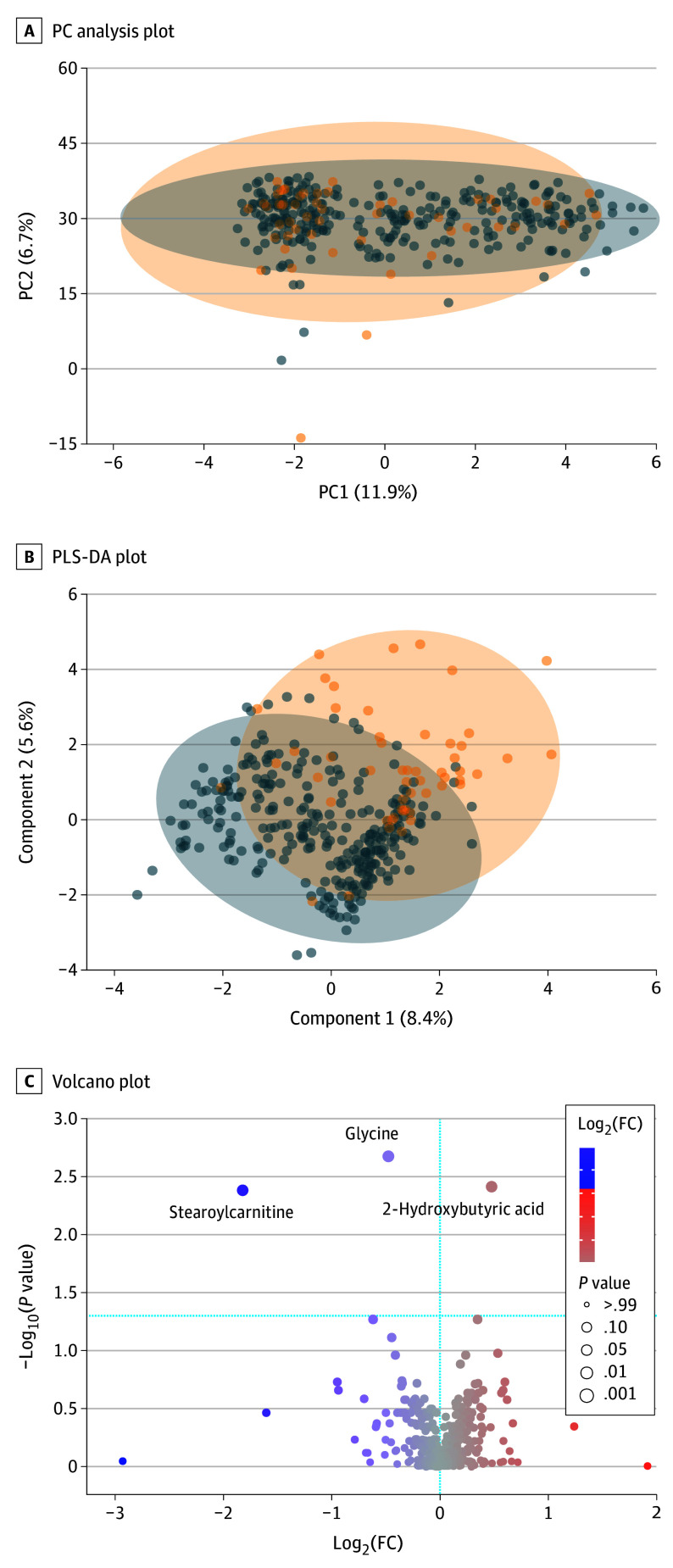
Metabolomic Analysis of Human Milk According to Gestational Diabetes Status Metabolomics data summary of A, the principal component (PC) analysis plot of milk metabolites by nongestational diabetes (GD) (0) and GD (1) status; B, partial least-squares discriminant analysis (PLS-DA) plot of milk metabolites by non-GD (0) and GD (1) status; and C, volcano plot of metabolites significantly associated with GD vs non-GD status with the horizontal line across the plot indicating the FDR-corrected *P* < 0.05 cutoff for statistical significance and the top 3 metabolites labeled. Orange circles represent GD group and blue circles represent non-GD. All figures created in MetaboAnalyst. FC indicates fold change.

**Figure 2.**
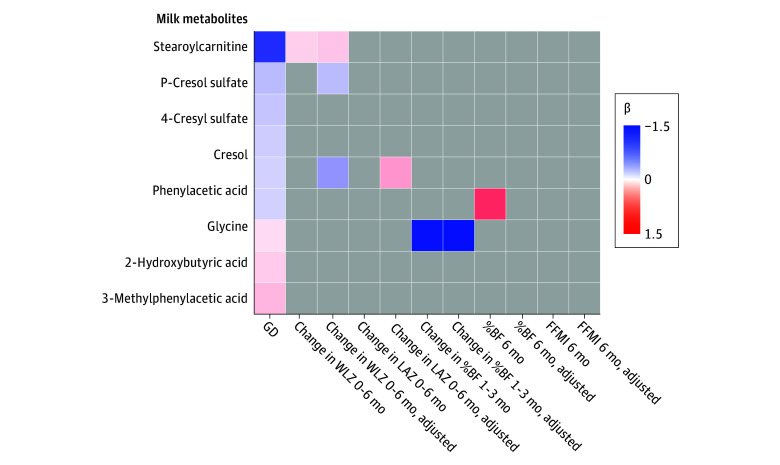
Association of Gestational Diabetes (GD)–Related Metabolites With Infant Growth and Body Composition Nine milk metabolites were significantly associated with maternal GD in covariate-adjusted models (FDR-corrected *P* < 0.05). The color of each cell represents the β coefficients in linear regression models. The first column is the association of each milk metabolite with maternal GD. Subsequent columns are β coefficients of association of each milk metabolite with infant body composition measures in unadjusted and adjusted models. Only significant associations (*P* < .05) are shown. Figure created in Morpheus (Broad Institute). %BF indicates percentage body fat; FFMI, fat-free mass index; LAZ, length for age *z* score; WLZ, weight for length *z* score.

A total of 458 metabolites were detected in human milk samples. Global separation by GD was not observed in principal component plots (Figure 1). In univariable regression models, GD was associated with 3 metabolites (glycine: β = −0.18; SE, 0.04; FDR, 0.002; 2-hydroxybutyric acid: β = 0.12; SE, 0.03; FDR = 0.004; stearoylcarnitine: β = −0.45; SE, 0.11; FDR = 0.004) (eTable 5 in Supplement 1). After covariate adjustment, GD was negatively associated with 6 metabolites, including an acylcarnitine (stearoylcarnitine), an amino acid (glycine), 2 arylsulfates (4-cresyl sulfate and p-cresol sulfate), and 2 benzenoids (phenylacetic acid and cresol) (eTable 6 in Supplement 1; Figure 2). GD was positively associated with 3 metabolites, including an alpha hydroxy acid (2-hydroxybutyric acid [2-HB]), benzenoid (3-methylphenylacetic acid), and a steroid (pregnanolone sulfate) (eTable 6 in Supplement 1; Figure 2). Pathway analysis revealed overrepresentation of metabolites belonging to glyoxylate and dicarboxylate metabolism (*P* = .002), citrate cycle (*P* = .01), porphyrin metabolism (*P* = .03), and valine, leucine, and isoleucine degradation (*P* = .04) metabolic pathways, but results were not statistically significant after FDR correction (eTable 7 in Supplement 1).

In univariable models, OGCT was associated with 2 metabolites, asparagine and benzoate (asparagine: β = −0.004; SE, 0.00; FDR = 0.002; benzoate: β = 0.003; SE, 0.001; FDR = 0.003) (eTable 8 in Supplement 1), and in adjusted models, 6 metabolites were associated (stearoylcarnitine: β = −0.008, SE, 0.002, FDR < 0.001; indole-3-carboxylic acid: β = −0.003; SE, 0.001; FDR = 0.02; asparagine: β = −0.003; SE, 0.001; FDR = 0.049; nonanedioate: β = 0.003; SE, 0.001; FDR = 0.01; pregnanolone sulfate: β = 0.002; SE, 0.001; FDR = 0.02; uridine: β = 0.002; SE, 0.001; FDR = 0.02). OGCT was negatively associated with stearoylcarnitine, indole-3-carboxylic acid, and asparagine and positively associated with nonanedioate, pregnanolone sulfate, and uridine (eTable 9 in Supplement 1).

### GD-Associated Milk Metabolites, OGCT, and Infant Growth and Body Composition

In adjusted analyses, 3 of the 9 milk metabolites that were significantly associated with GD were also associated with infant growth and body composition measures: 2-HB was negatively associated with change in %BF from 1 to 3 months (β = −1.50; SE, 0.66; 95% CI, −2.79 to −4.82; *P* = .03) (eTable 10 in Supplement 1), phenylacetic acid was positively associated with change in LAZ score from 0 to 6 months (β = 0.27; SE, 0.13; 95% CI, 0.02 to 0.16; *P* = .04) and stearoylcarnitine (odds ratio, 1.65; 95% CI, 1.23 to 2.25) was associated with greater odds of RWG (eTable 11 in Supplement 1).

In unadjusted and adjusted analyses, infants exposed to GD had greater change in LAZ from 0 to 6 months (adjusted analysis: β = 0.48; SE, 0.22; 95% CI, 0.05 to 0.32; *P* = .03) than infants of participants without GD, but no other associations between GD and growth or body composition measures were found (eTable 13 in Supplement 1). OGCT was not significantly associated with infant growth or body composition (eTable 12 in Supplement 1).

## Discussion

In an exclusively breastfeeding cohort, we aimed to identify differences in the human milk metabolome according to maternal GD diagnosis. We also assessed the association between metabolites that were significantly associated with GD and infant growth and body composition measures. Finally, we assessed whether GD was associated with differences in infant growth or body composition. We found that GD was associated with differences in the relative abundance of 9 human milk metabolites. Three of these metabolites were also associated with infant growth and body composition. The metabolite 2-HB, which was positively associated with GD, was negatively associated with the change in infant %BF from 1 to 3 months, while phenylacetic acid, which was negatively associated with GD, was positively associated with change in infant LAZ score from 0 to 6 months. Stearoylcarnitine, which was negatively associated with GD, was associated with greater odds of RWG from 0 to 6 months. While others have found GD to be associated with increased adiposity,^28^ we did not observe significant differences in growth or adiposity between groups, except for greater linear growth (change in LAZ from 0-6 months) for the GD group. We also noted that maternal OGCT was associated with decreased milk stearoylcarnitine and increased pregnanolone sulfate, providing further support that the metabolite differences may stem from differences in glucose regulation. Taken together, our data show that GD is associated with differences in the abundance of several human milk metabolites, and that a subset of these milk components, in turn, are associated with infant growth and body composition. However, the direction of the associations does not suggest an adverse effect of GD-associated changes on infant adiposity, nor on at-risk patterns of accelerated postnatal weight gain. Rather, the directionality suggests that milk compositional differences in GD mothers may subtly, but beneficially, moderate the growth of their infants.

We are aware of only 2 prior studies, both conducted in Chinese populations, that examined GD-associated changes in the human milk metabolome. In a sample of 90 participants with GD and 94 participants without GD, Wen et al^15^ found differences in abundance of 28 metabolites in colostrum, transitional, and mature milk, including differences in 20 metabolites (5 metabolites higher, 15 metabolites lower; *q* value < .10) at 4 weeks post partum. Despite geographical differences and use of different metabolomics platforms, 3 of the metabolites identified by Wen et al^15^ showed similar associations in our cohort (eTable 6 in Supplement 1).

Another study by Wu et al^16^ conducted untargeted metabolomic analysis of human milk in 100 participants (50 participants with GD and 50 without). At day of life (DOL) 42, 28 metabolites differed between the GD and non-GD groups. Three of the metabolites (2-pyrrolidinone, malate, and pantothenate) identified by Wu et al^16^ were associated with GD in our cohort (eTable 6 in Supplement 1). Wu et al^16^ also examined differences in infant growth trajectories over the first year of life but did not assess body composition. In contrast with our findings, they did not observe associations between human milk metabolites and linear growth rates, but they did report increased infant length at 10 days of life in the GD group. However, GD-exposed infants had greater weight gain from DOL 42 to 1 year of age, which contrasts with our results. These differences may potentially stem from methodological differences between our studies, including differences in covariate adjustment and duration of follow-up.

In our analysis, the 3 metabolites that were associated with both GD and infant growth or body composition (ie, stearoylcarnitine, 2-HB, and phenylacetic acid) have previously been linked to lipid metabolism, GD, or insulin resistance. While the mechanisms responsible for the observed differences in the human milk metabolome have yet to be elucidated, GD is characterized by metabolic changes such as increased insulin resistance, decreased insulin production, and alterations in lipid metabolism, which may potentially influence human milk composition.^29^ We found that milk stearoylcarnitine was lower in GD, and was associated with higher odds of rapid weight gain. Acylcarnitines such as stearoylcarnitine are involved in fatty acid transport into the mitochondria for β oxidation, and elevated concentrations may suggest incomplete fatty acid oxidation.^30^ Higher serum acylcarnitines have also been associated with insulin resistance and type 2 diabetes.^31^ We found that abundance of 2-HB, a metabolite linked to lipid metabolism, was higher in participants with GD and negatively associated with the change in infant %BF from 1 to 3 months. This alpha-hydroxy acid is produced from the catabolism of threonine and methionine and previously has been identified in plasma from patients in insulin resistance states, including in a study of insulin resistance during pregnancy.^32,33^ In animal models, 2-HB has been observed to modulate lipid metabolism, perhaps explaining the association between milk concentrations and decreased adiposity.^32^ Moreover, we found that GD was associated with decreased abundance of phenylacetic acid; this benzenoid metabolite was positively associated with the change in infant LAZ from 0 to 6 months. Interestingly, higher levels of phenylacetate in gut microbiota and in serum have been linked to GD.^35,36^ In animal studies, phenylacetic acid has been shown to decrease glucose-stimulated insulin secretion by inhibiting pyruvate carboxylase.^34^ The relative abundances of these 3 metabolites in milk were opposite the serum or gut concentrations reported in the literature for participants with GD. We did not analyze maternal serum metabolites; thus, we are unable to determine whether the abundances of these milk metabolites were reflective of maternal serum levels. Additionally, we do not know the fate of these metabolites after ingestion by the infant or whether differences in their abundances contributed, at a mechanistic level, to growth and body composition patterns. While our study identified candidate metabolites potentially involved in the relationship between GD and infant growth, further work is needed to better understand their role in infant health.

The growth and adiposity patterns observed for infants exposed to GD in our study were in contrast to other studies, which have found increased birth weight and adiposity for GD-exposed infants.^37,38^ Our findings may be due to differences between our cohort and other published GD cohorts. In comparison with other studies,^39,40^ all participants with GD in our study were exclusively breastfeeding at 1 month post partum, had received treatment for GD, were mostly White, and the majority were upper to middle class. While excessive maternal gestational weight gain has been linked to offspring of higher birth weights (large for gestational age),^39^ our group with GD had lower gestational weight gain compared with the group without. We speculate that contemporary women with GD who elect to and are able to exclusively breastfeed might differ from the older body of literature on GD, perhaps related to differences in socioeconomics, disease management, or other characteristics. Some groups have suggested that certain subtypes of GD are less likely to lead to fetal overgrowth,^41^ and others have found that infants exposed to GD had lower birth weight and that GD was not a significant predictor of neonatal adiposity at birth.^42^ Overall, our findings add to the growing literature on the heterogeneity of GD and reinforce the benefits of breastfeeding for all mother-infant dyads.^43^

### Limitations and Strengths

We acknowledge several study limitations. First, the use of the 1982 diagnostic criteria for GD may have influenced our results. The new diagnostic criteria have resulted in increasing the incidence of GD by more than 2-fold.^44^ Accordingly, it is possible that more of our participants would have been formally diagnosed with GD and undergone treatment if the new criteria had been applied. Because the groups would likely have become more similar, this could result in bias toward the null hypothesis (ie, making it more likely to find no differences in milk metabolites between groups with and without GD). Second, we do not have information about participants’ postpartum glucose metabolism and could not assess GD resolution, nor postpartum insulin resistance and/or hyperglycemia. We were also unable to assess adherence to treatment for GD. Third, we did not analyze maternal serum metabolite levels, preventing us from determining whether alterations in milk metabolites stem from differences in circulating levels. Moreover, our cohort was predominately White and highly educated, limiting generalizability of our findings. We acknowledge that while maternal dietary quality scores were not different by GD status, individual dietary components may have shaped the milk metabolome. However, a detailed examination of these components is beyond the scope of this study. Also, our milk analysis was limited to 1 month samples (mature milk), and the infant follow-up visits were limited to 6 months, preventing us from determining changes in the milk metabolome or infant growth beyond these time points. Additionally, our study was observational and we are unable to infer causality. Future studies will be essential to determine whether metabolites associated with GD may alter metabolic physiology in the infant, and whether they act within the gut or other tissues.

Despite these limitations, we report differences in the milk metabolome by GD status in the largest US-based cohort we know of to date. As an additional strength, our study collected extensive data on maternal and infant characteristics and included a rigorous protocol for obtaining human milk samples in a research setting. A large sample size allowed us to account for multiple testing and adjust for many covariates and potential confounders that may affect milk composition.

## Conclusions

In this US-based study of exclusively breastfeeding mother-infant dyads, we found 9 human milk metabolites (out of more than 400) that significantly differed by GD status, 3 of which were associated with faster linear growth, lower adiposity gain, or lower risk of rapid weight gain. This finding highlights the potential role of breastfeeding among women with GD in moderating offspring obesity risk, and reinforces the benefits of breastfeeding for all parents and infants.

## Notes

Supplement 1. **eMethods 1.** Supplemental Information on metabolomic analysis
eReferences.
**eFigure 1.** Directed Acyclic Graph of GD and human milk metabolites **eFigure 2.** Directed Acyclic Graph of milk metabolites and infant growth and body composition outcomes **eTable 1.** %CV of all metabolites in quality control technical replicates **eTable 2.** Non-GD participant characteristics with P values (n=395) **eTable 3.** Missing data from table 1 **eTable 4.** Participant characteristics with P values (n=348) **eTable 5.** Unadjusted associations between maternal gestational diabetes status and 1-month human milk metabolites n=348 **eTable 6.** Adjusted associations between maternal GD status and 1-month human milk metabolites n=322 **eTable 7.** Pathway Enrichment Analysis **eTable 8.** Unadjusted associations between maternal OGCT results and 1-month human milk metabolites n=270 **eTable 9.** Adjusted associations between maternal OGCT results and 1-month human milk metabolites n=270 **eTable 10.** Associations between milk metabolites and infant growth and body composition **eTable 11.** Associations between milk metabolites associated with GD and rapid infant weight gain **eTable 12. **Associations between OGCT and infant growth and body composition **eTable 13. **Associations between GD and infant body composition and growth variables

Supplement 2.
Data Sharing Statement
