# Error in Funding

**DOI:** 10.1001/jamanetworkopen.2024.59711

**Published:** 2025-01-10

In the Original Investigation titled “Gestational Diabetes, the Human Milk Metabolome, and Infant Growth and Adiposity,”^1^ published December 12, 2024, there was an error in the Funding disclosure. Dr Nagel was supported by a National Institutes of Health/Eunice Kennedy Shriver National Institute of Child Health and Human Development grant, No. R00HD108276, which was not listed. This article has been corrected.^1^

## References

[1] Nagel EM, Peña A, Dreyfuss JM, . Gestational diabetes, the human milk metabolome, and infant growth and adiposity. JAMA Netw Open. 2024;7(12):e2450467. doi:10.1001/jamanetworkopen.2024.50467 39666338 PMC11638796

