# Supplementary material for: Gestational Diabetes, the Human Milk Metabolome, and Infant Growth and Adiposity
Source: JAMA Netw Open. 2024 Dec 12;7(12):e2450467. doi: 10.1001/jamanetworkopen.2024.50467 (PMC11638796; doi:10.1001/jamanetworkopen.2024.50467)
Supplement: Supplement 1. — eMethods 1. Supplemental Information on metabolomic analysis eReferences. eFigure 1. Directed Acyclic Graph of GD and human milk metabolites eFigure 2. Directed Acyclic Graph of milk metabolites and infant growth and body composition outcomes eTable 1. %CV of all metabolites in quality control technical replicates eTable 2. Non-GD participant characteristics with P values (n=395) eTable 3. Missing data from table 1 eTable 4. Participant characteristics with P values (n=348) eTable 5. Unadjusted associations between maternal gestational diabetes status and 1-month human milk metabolites n=348 eTable 6. Adjusted associations between maternal GD status and 1-month human milk metabolites n=322 eTable 7. Pathway Enrichment Analysis eTable 8. Unadjusted associations between maternal OGCT results and 1-month human milk metabolites n=270 eTable 9. Adjusted associations between maternal OGCT results and 1-month human milk metabolites n=270 eTable 10. Associations between milk metabolites and infant growth and body composition eTable 11. Associations between milk metabolites associated with GD and rapid infant weight gain eTable 12. Associations between OGCT and infant growth and body composition eTable 13. Associations between GD and infant body composition and growth variables [file jamanetwopen-e2450467-s001.pdf]

Supplemental Online Content

Nagel EM, Peña A, Dreyfuss JM, et al. Gestational diabetes, the human milk metabolome, and infant growth and adiposity. *JAMA Netw Open*. 2024;7(12):e2450467. doi:10.1001/jamanetworkopen.2024.50467

eMethods 1. Supplemental Information on metabolomic analysis
eReferences.
eFigure 1. Directed Acyclic Graph of GDM and human milk metabolites
eFigure 2. Directed Acyclic Graph of milk metabolites and infant growth and body composition outcomes
eTable 1. %CV of all metabolites in quality control technical replicates
eTable 2. Non-GDM participant characteristics with P values (n=395)
eTable 3. Missing data from table 1
eTable 4. Participant characteristics with P values (n=348)
eTable 5. Unadjusted associations between maternal gestational diabetes status and 1-month human milk metabolites n=348
eTable 6. Adjusted associations between maternal GDM status and 1-month human milk metabolites n=322
eTable 7. Pathway Enrichment Analysis
eTable 8. Unadjusted associations between maternal OGCT results and 1-month human milk metabolites n=270
eTable 9. Adjusted associations between maternal OGCT results and 1-month human milk metabolites n=270
eTable 10. Associations between milk metabolites and infant growth and body composition
eTable 11. Associations between milk metabolites associated with GDM and rapid infant weight gain
eTable 12. Associations between OGCT and infant growth and body composition
eTable 13. Associations between GDM and infant body composition and growth variables

This supplemental material has been provided by the authors to give readers additional information about their work.

© 2024 Nagel EM et al. *JAMA Network Open.*

eMethods 1. Supplemental Information on metabolomic analysis

Human milk samples for metabolomics analysis were prepared per previously described protocols [1-5].

Metabolite extraction
A mixture of isopropanol, acetonitrile, and water at a ratio of 3:3:2 v/v were utilized. Prior to analysis, extracts were divided in to three parts: 75 uL (gas chromatography combined with time-of-flight high-resolution mass spectrometry), 150 uL (reversed-phase liquid chromatography coupled with high-resolution mass spectrometry), and 150 uL (hydrophilic interaction chromatography with liquid chromatography and tandem mass-spectrometry).[1–5]

Hydrophilic interaction liquid chromatography analysis
The NEXERA XR UPLC system (Shimadzu, Columbia, MD, USA) coupled with the Triple Quad 5500 System (AB Sciex, Framingham, MA, USA) was used.

Reversed-phase liquid chromatography analysis
The NEXERA XR UPLC system (Shimadzu, Columbia, MD, USA) coupled with the Triple TOF 6500 System (AB Sciex, Framingham, MA, USA) and Agilent 7890B gas chromatograph (Agilent, Palo Alto, CA, USA) interfaced to a Time-of-Flight Pegasus HT Mass Spectrometer (Leco, St. Joseph, MI, USA) was used. The GC system fitted with a Gerstel temperature-programmed injector, cooled injection system (model CIS 4). To eliminate cross-contamination from the sample matrix occurring between sample runs, an automated liner exchange (ALEX) (Gerstel, Muhlheim an der Ruhr, Germany) was used.

Quality Control
Conducted using metabolite standards mixture, external pooled human plasma, and pooled milk samples applying methodology per previous description.[6–9] To assess analytical system suitability tests, a quality control sample containing a standard mixture of amino and organic acids (Sigma-Aldrich) as certified reference material was injected daily. Monitor recorded signals day to day reproducibility as described previously.[1–4,10] External human plasma samples (Sigma-Aldrich) were injected after each tenth sample in a batch. A pooled quality control sample was obtained by taking an aliquot of the same volume of all samples from the study and injected daily with a batch of analyzed samples to determine the optimal dilution of the batch samples and validate metabolite identification and peak integration. Collected raw data was manually inspected, merged, and imputed. We performed batch correction was using COMBAT protocols.

Metabolite identification
Conducted using in house authentic standards analysis. Metabolite annotation conducted utilizing recorded retention time and retention indexes, recorded MS^n and $HRAMS^n$ data matching with the following databases (METLIN, NIST MS, Wiley Registry of Mass Spectral Data, HMDB, MassBank of North America, MassBank Europe, Golm Metabolome Database, SCIEX Accurate Mass Metabolite Spectral Library, MzCloud, and IDEOM).

© 2024 Nagel EM et al. *JAMA Network Open.*

eReferences.

1. Tolstikov V, Nikolayev A, Dong S, Zhao G, Kuo MS. Metabolomics Analysis of Metabolic Effects of Nicotinamide Phosphoribosyltransferase (NAMPT) Inhibition on Human Cancer Cells. *PLoS One*. 2014;9(12):e114019. doi:10.1371/JOURNAL.PONE.0114019
2. Ntranos A, Park HJ, Wentling M, et al. Bacterial neurotoxic metabolites in multiple sclerosis cerebrospinal fluid and plasma. *Brain*. 2022;145(2):569-583. doi:10.1093/BRAIN/AWAB320
3. Baskin AS, Linderman JD, Brychta RJ, et al. Regulation of Human Adipose Tissue Activation, Gallbladder Size, and Bile Acid Metabolism by a β3-Adrenergic Receptor Agonist. *Diabetes*. 2018;67(10):2113-2125. doi:10.2337/DB18-0462
4. Drolet J, Tolstikov V, Williams BA, et al. Integrated Metabolomics Assessment of Human Dried Blood Spots and Urine Strips. *Metabolites*. 2017;7(3). doi:10.3390/METABO7030035
5. Kiebish MA, Cullen J, Mishra P, et al. Multi-omic serum biomarkers for prognosis of disease progression in prostate cancer. *J Transl Med*. 2020;18(1):10. doi:10.1186/S12967-019-02185-Y
6. Dunn WB, Broadhurst D, Begley P, et al. Procedures for large-scale metabolic profiling of serum and plasma using gas chromatography and liquid chromatography coupled to mass spectrometry. *Nat Protoc*. 2011;6(7):1060-1083. doi:10.1038/NPROT.2011.335
7. Bajad S, Shulaev V. LC-MS-based metabolomics. *Methods Mol Biol*. 2011;708:213-228. doi:10.1007/978-1-61737-985-7_13
8. Yuan M, Breitkopf SB, Yang X, Asara JM. A positive/negative ion-switching, targeted mass spectrometry-based metabolomics platform for bodily fluids, cells, and fresh and fixed tissue. *Nat Protoc*. 2012;7(5):872-881. doi:10.1038/NPROT.2012.024
9. Want EJ, Masson P, Michopoulos F, et al. Global metabolic profiling of animal and human tissues via UPLC-MS. *Nat Protoc*. 2013;8(1):17-32. doi:10.1038/NPROT.2012.135
10. Kiebish MA CJMPAAMERLCETVZLPKSPCYPGRISIMDGESRAVSASSNNDA. Multi-omic serum biomarkers for prognosis of disease progression in prostate cancer. *J Transl Med*. 2020;18(1):10. doi:10.1186/S12967-019-02185-Y

© 2024 Nagel EM et al. *JAMA Network Open.*

eFigure 1. Directed Acyclic Graph of GDM and human milk metabolites

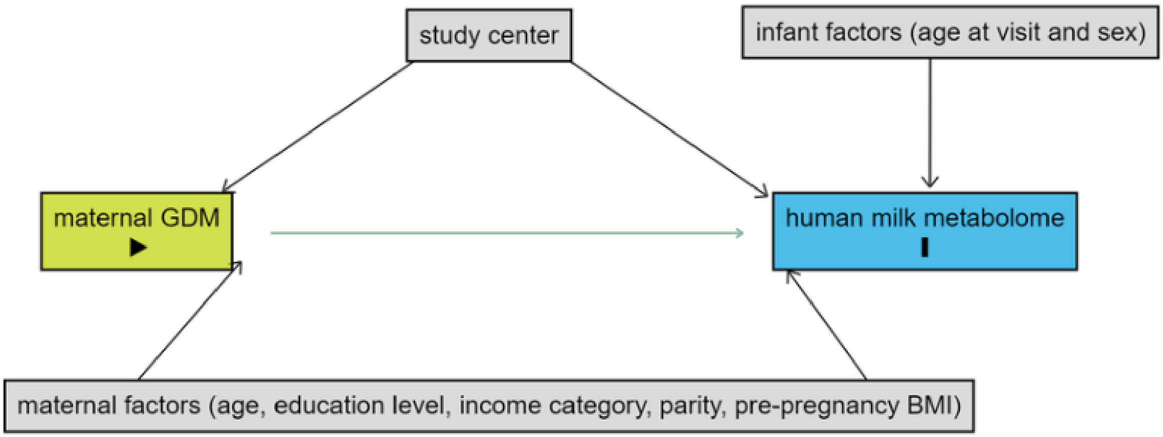

Directed acyclic graph modeling the exposure (maternal GDM) and outcome (human milk metabolome) with potential confounders (study center and maternal factors) and covariates (infant factors).

© 2024 Nagel EM et al. *JAMA Network Open.*

eFigure 2. Directed Acyclic Graph of milk metabolites and infant growth and body composition outcomes

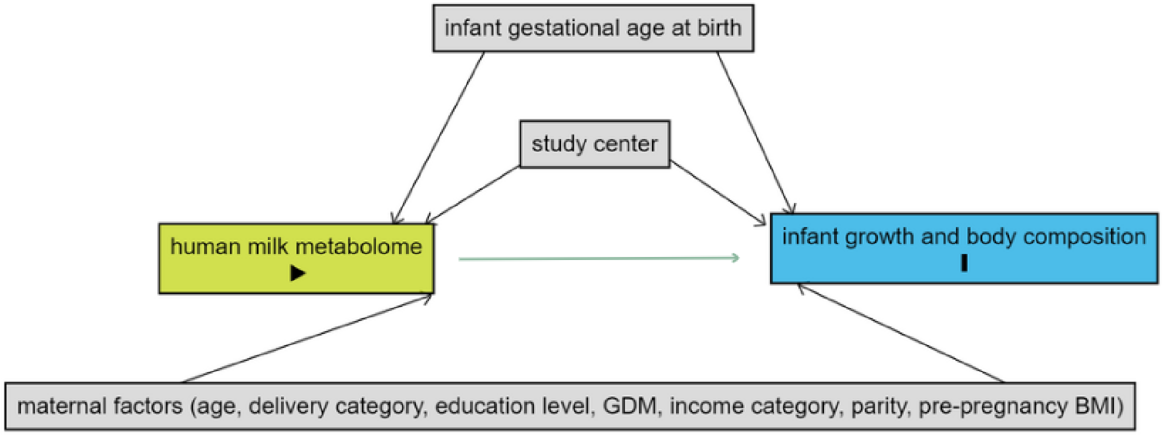

Directed acyclic graph modeling the exposure (human milk metabolome) and outcome (infant growth and body composition) with potential confounders (infant gestational age at birth, study center, maternal factors). Infant body composition models additionally adjusted for covariates of infant age at visit and sex.

© 2024 Nagel EM et al. *JAMA Network Open.*

eTable 1. %CV of all metabolites in quality control technical replicates

Metabolite	%CV (log transformed)
(2E,5Z,7E)-DECATRIENOYLCARNITINE	36%
(4-ACETYL-2-METHOXYPHENYL)OXIDANESULFONIC ACID	42%
1,3-DIMETHYLURIC ACID	138%
1,3-PROPANEDIOL	47%
1,5-ANHYDROSORBITOL	27%
1-HEXADECYL-GLYCERO-3-PHOSPHATE	41%
1H-Indole-3-carboxaldehyde	42%
1-METHYLADENINE	23%
1-METHYLADENOSINE	4%
1-METHYLHISTAMINE	66%
1-METHYL-HISTIDINE	4%
1-METHYLNICOTINAMIDE	6%
1-PALMITOYL-SN-GLYCERO-3-PHOSPHOCHOLINE	3%
2,3-DIHYDROXYBENZOIC ACID	48%
2,8-DIHYDROXYADENINE	25%
2-AMINOADIPIC ACID	28%
2-AMINOETHYLPHOSPHONATE	65%
2-AMINOISOBUTYRATE	8%
2-AMINOOCTANOIC ACID	4%
2-DEHYDRO-D-GLUCONATE	48%
2-FUROIC ACID	8%
2-FUROYLGLYCINE	6%
2-HYDROXY-3-METHYLBUTYRIC ACID	13%
2-HYDROXYBUTYRIC ACID	8%
2-HYDROXYGLUTARATE	25%
2-HYDROXYHEXANOATE	9%
2-HYDROXYVALERATE	5%
2-ISOPROPYLMALIC ACID	128%
2-KETOHEXANOIC ACID	61%
2-METHOXY-PHENOL	24%

© 2024 Nagel EM et al. *JAMA Network Open.*

2-METHYL-3-KETOVALERIC ACID	24%
2-METHYLBUTYROYLCARNITINE	5%
2-METHYLGLUTARIC ACID	6%
2-NONENOYLCARNITINE	10%
2-OCTANDIOIC-CARNITINE	26%
2-OCTENDIOIC-CARNITINE	58%
2-OCTENOYLCARNITINE	4%
2-OXO-4-METHYLTHIOBUTANOATE	79%
2-OXOBUTANOATE	15%
2-OXOGLUTARATE	98%
2-PHENYLGLYCINE	18%
2-PHENYLLACTIC ACID	21%
2-PYRROLIDINONE	4%
3,4-DIHYDROXYPHENYLACETIC ACID	41%
3-AMINOBUTYRATE	60%
3-AMINOISOBUTANOIC ACID	68%
3-CARBOXY-4-METHYL-5-PROPYL-2-FURANPROPIONATE	80%
3-DEHYDROXYCARNITINE	4%
3-HYDOXYPHENYLACETIC ACID	24%
3-HYDROXY-3-METHYLGLUTARYLCARNITINE	37%
3-HYDROXYBUTYRIC ACID	14%
3-HYDROXYDODECANOIC ACID	21%
3-HYDROXYHEXADECANOATE	16%
3-HYDROXYISOBUTYRATE	19%
3-Hydroxyisovalerylcarnitine	6%
3-HYDROXY-MYRISTOYL-CARNITINE	32%
3-HYDROXYOCTANOYLCARNITINE	34%
3-HYDROXYSUBEROYLCARNITINE	49%
3-INDOLE-ACETIC ACID	176%
3-KETOSUBEROYLCARNITINE	61%
3-METHOXYBENZENEPROPANOIC ACID	43%
3-METHYL-2-OXOVALERIC ACID	21%

© 2024 Nagel EM et al. *JAMA Network Open.*

3-METHYLGLUTARYLCARNITINE	21%
3-METHYLPHENYLACETIC ACID	36%
3-OXOOCTADECANOATE	19%
3-PHOSPHOGLYCERATE	33%
4-CRESYL SULFATE	9%
4-GUANIDINOBUTANOATE	13%
4-HYDROXYBENZOIC ACID	24%
4-HYDROXYPHENYLLACTIC ACID	17%
4-HYDROXYPHENYLPYRUVATE	48%
4-OXODECANEDIOATE	62%
4-OXOPROLINE	53%
4-PYRIDOXIC ACID	10%
5,6-DIHYDRO-5-METHYLURACIL	63%
5-AMINO-3-OXOHEXANOIC ACID	17%
5-AMINOVALERIC ACID	31%
5-HYDROXYINDOLE-3-ACETIC ACID	60%
5-HYDROXYINDOLEACETIC ACID	36%
5-HYDROXY-L-TRYPTOPHAN	53%
5-HYDROXYLYSINE	51%
5-HYDROXYMETHYL-2-FUROYLCARNITINE	4%
5-METHOXYTRYPTOPHAN	82%
5-METHYLTETRAHYDROFOLATE	86%
5-OXOPROLINE	69%
6-HYDROXYDOPAMINE	84%
6-KETO-DECANOYLCARNITINE	67%
7-METHYLGUANINE	10%
7-METHYLGUANOSINE	30%
8-HYDROXY-2-DEOXYGUANOSINE	71%
9-DECENOYLCARNITINE	29%
ACETOACETATE	71%
ACETYLCARNITINE	3%
ACETYLCHOLINE	3%

© 2024 Nagel EM et al. *JAMA Network Open.*

ACETYLCYSTEINE	39%
ACETYLHISTAMINE	77%
ACETYLPHOSPHATE	90%
ACONITATE	40%
ADENINE	32%
ADENOSINE	63%
ADIPATE	68%
ADP	38%
ADP-RIBOSE-CYCLIC PHOSPHATE	57%
AICA RIBOSIDE	32%
AICAR	45%
AICAR-CYCLIC-PHOSPHATE	76%
ALANINE	4%
ALLANTOATE	27%
ALLANTOIN	29%
ALPHA-KETOISOVALERIC ACID	44%
AMP	13%
ANSERINE	20%
ANTHRANILATE	28%
ARABINITOL	102%
ARACHIDONATE	17%
ARGININE	7%
ASCORBIC ACID	55%
ASPARAGINE	7%
ASPARTATE	21%
ASSN,N-DIMETHYL-L-ARGININE	77%
ATP	83%
BENZOATE	5%
BENZOYLCARNITINE	13%
BETA-CITRYL-L-GLUTAMIC ACID	69%
BETA-GUANIDINOPROPIONATE	11%
BETAINE	3%

© 2024 Nagel EM et al. *JAMA Network Open.*

BETAINE ALDEHYDE	14%
BETA-N-ACETYLGLUCOSAMINE	90%
BILIRUBIN	40%
BIOTIN	66%
BUTYRYLCARNITINE	3%
CARBAMOYL PHOSPHATE	42%
CARNITINE	3%
CARNOSINE	69%
CDP-CHOLINE	82%
CDP-ETHANOLAMINE	84%
CHOLATE	21%
CHOLESTERYL SULFATE	24%
CHOLINE	4%
CINNAMOYL-GLUCOSIDE	101%
CITRAMALATE	66%
CITRATE	5%
CITRIC ACID	23%
CITRULLINE	6%
CMP	49%
CORTISOL	48%
COUMARIC ACID	7%
CREATINE	5%
CREATININE	3%
CRESOL	5%
CYCLIC-AMP	15%
CYSTATHIONINE	52%
CYSTEINE	60%
CYSTEINE-GLYCINE	73%
CYSTINE	16%
CYTIDINE	114%
CYTOSINE	15%
DAMP	36%

© 2024 Nagel EM et al. *JAMA Network Open.*

DCMP	81%
DECANEDIOATE	20%
DECANOATE	60%
DECANOYLCARNITINE	4%
DECENOYLCARNITINE	4%
DEHYDROCARNITINE	4%
DEOXYADENOSINE	100%
DEOXYCHOLATE	21%
DEOXYCYTIDINE	74%
DEOXYGUANOSINE	101%
DEOXYINOSINE	96%
DEOXYURIDINE	77%
D-ERYTHROSE-4-PHOSPHATE	78%
D-GLUCARATE	63%
D-GLUCONATE	48%
D-GLUCOSAMINE-6-PHOSPHATE	74%
D-Glyceraldehyde 3-phosphate	61%
DHA	78%
DHEA SULFATE	16%
DIHODE	7%
DIHYDROOROTATE	48%
DIHYDROURACIL	106%
DIHYDROXY-ACETONE-PHOSPHATE	94%
DIHYDROXYCHOLESTANOYL TAURINE	16%
DIHYDROXYPHENYLALANINE	24%
DIMETHYLGLYCINE	3%
DIMETHYLSULFONE	5%
DOCOSAHEXAENOATE	40%
DOCOSATRIENOIC ACID	21%
DOCOSENAMIDE	26%
DODECANEDIOIC ACID	18%
DODECANOATE	48%

© 2024 Nagel EM et al. *JAMA Network Open.*

DODECANOYLCARNITINE	9%
DOPAMINE	29%
D-RIBOSE 5-PHOSPHATE	57%
D-SEDOHEPTULOSE 7-PHOSPHATE	79%
EICOSENOATE	12%
EPINEPHRINE	164%
ETHANOLAMINE	15%
ETHYLMALONIC ACID	74%
FAD	34%
FAPY-ADENINE	67%
FLAVONE	64%
FOLATE	86%
FRUCTOSE	26%
FRUCTOSE-6-PHOSPHATE	81%
FUCOSE	10%
FUMARATE	24%
FURANEDICARBOXYLCARNITINE	45%
GALACTOSE	24%
GALACTOSYLHYDROXYLYSINE	57%
GAMMA-GLUCYS	57%
GAMMA-GLU-GLN	9%
GAR	16%
GLU-ALA	67%
GLUCONIC ACID LACTONE	83%
Gluconolactone	65%
GLUCOSAMINE	23%
GLUCOSE	24%
GLUCOSE-1,6-BISPHOSPHATE	97%
GLUCOSE-1-PHOSPHATE	56%
GLUCOSE-6-PHOSPHATE	98%
GLUCOSYLGALACTOSYLHYDROXYLYSINE	56%
GLU-LEU	21%

© 2024 Nagel EM et al. *JAMA Network Open.*

GLUTACONIC ACID	51%
GLUTACONYLCARNITINE	31%
GLUTAMATE	6%
GLUTAMIC ACID	34%
GLUTAMINE	4%
GLUTARIC ACID	82%
GLUTARYLCARNITINE	20%
GLUTATHIONE	67%
GLYCERATE	72%
GLYCEROL	28%
GLYCEROL-3-PHOSPHATE	36%
GLYCEROPHOSPHOCHOLINE	3%
GLYCERYLPHOSPHORYLETHANOLAMINE	17%
GLYCINE	32%
GLYCOLATE	49%
GLYOXYLATE	84%
GMP	40%
GUANIDINEACETIC ACID	39%
GUANIDINOSUCCINIC ACID	26%
GUANINE	92%
GUANOSINE	105%
HEPTADECANOATE	59%
HEPTANOYLCARNITINE	11%
HEXADECANEDIOATE	32%
HEXANOIC ACID	82%
HEXANOYLCARNITINE	6%
HIPPURIC ACID	80%
HISTIDINE	5%
HISTIDINOL	26%
HOMOCARNOSINE	55%
HOMOCITRULLINE	73%
HOMOCYSTEIC ACID	76%

© 2024 Nagel EM et al. *JAMA Network Open.*

HOMOCYSTEINE	83%
HOMOSERINE	34%
HOMOVANILIC ACID	109%
HYDROXYBUTYRYLCARNITINE	6%
HYDROXYISOCAPROIC ACID	9%
HYDROXYPHENYLACETIC ACID	43%
Hydroxyphenylacetylglycine	28%
HYDROXYPHENYLLACTIC ACID	54%
HYDROXYPROLINE	7%
HYPOXANTHINE	5%
HYPUSINE	46%
IDP	57%
IMIDAZOLE	54%
IMIDAZOLEACETIC ACID	10%
IMIDAZOLEPROPIONIC ACID	38%
IMP	23%
INDOLE	38%
INDOLE-3-CARBOXYLIC ACID	40%
INDOLEACRYLIC ACID	12%
INDOXYL	11%
INDOXYL SULFATE	4%
INOSINE	87%
ISOBUTYRYLGLYCINE	59%
ISOBUTYRYL-L-CARNITINE	66%
ISOCITRATE	20%
ISOLEUCINE	3%
KYNURENIC ACID	32%
KYNURENINE	16%
LACTATE	6%
LACTIC ACID	75%
LACTOSE	74%
LACTOSE-PHOSPHATE	95%

© 2024 Nagel EM et al. *JAMA Network Open.*

L-ARGININO-SUCCINATE	73%
LEUCINE	4%
LINOLATE	18%
LINOLENATE	22%
LYSINE	9%
MALATE	12%
MALEIC ACID	18%
MALONYLCARNITINE	31%
MALTITOL	104%
MANDELOYLCARNITINE	11%
METHIONINE	9%
METHIONINE SULFOXIDE	18%
METHYL SUCCINIC ACID	55%
METHYLIMIDAZOLEACETIC ACID	7%
METHYLMALONIC ACID	36%
METHYLMALONYLCARNITINE	29%
MEVALONATE	55%
MEVALONATE-5-PHOSPHATE	60%
MYO-INOSITOL	12%
MYO-INOSITOL 1-PHOSPHATE	65%
MYRISTOYLCARNITINE	17%
N,N-DIMETHYL-L-ARGININE	6%
N1-ACETYLSPERMIDINE	77%
N2,N2-DIMETHYLGUANOSINE	16%
N2-METHYLGUANOSINE	32%
N4-ACETYLCYTIDINE	13%
N6-ACETYL-L-LYSINE	16%
N-ACETYL_L-TYROSINE	63%
N-ACETYLALANINE	68%
N-ACETYLASPARAGINE	136%
N-ACETYLASPARTIC ACID	28%
N-ACETYLCARNOSINE	13%

© 2024 Nagel EM et al. *JAMA Network Open.*

N-ACETYL-GLUCOSAMINE-1-PHOSPHATE	54%
N-ACETYL-GLUTAMATE	26%
N-ACETYLGLUTAMINE	34%
N-ACETYL-L-TYROSINE	69%
N-ACETYL-NEURAMINIC ACID	19%
N-ACETYLORNITHINE	58%
N-ACETYLPUTRESCINE	20%
N-ACETYLSERINE	17%
NAD+	67%
NADH	76%
NADP+	45%
NADPH	62%
N-ALPHA-ACETYLARGININE	15%
N-CARBAMOYL-L-ASPARTATE	70%
N-FORMYL-GAR	106%
N-FORMYLGLYCINE	67%
N-FORMYL-L-METHIONINE	33%
NICOTINAMIDE	12%
NICOTINAMIDE RIBOTIDE	36%
NICOTINATE	54%
NONANEDIOATE	61%
NONANOYLCARNITINE	8%
NOREPINEPHRINE	221%
N-PHENYLACETYL-GLUTAMINE	6%
O-ACETYL-L-SERINE	24%
OCTADECADIENAL	16%
OCTANOATE	21%
OCTANOYLCARNITINE	4%
OCTOPINE	61%
OLEAMIDE	29%
OLEATE	16%
OLEOYLCARNITINE	13%

© 2024 Nagel EM et al. *JAMA Network Open.*

O-PHOSPHORYLETHANOLAMINE	38%
ORNITHINE	11%
OROTATE	67%
OROTIDINE-5-PHOSPHATE	70%
OXALATE	59%
PALMITAMIDE	25%
PALMITATE	26%
PALMITOLINOLEATE	39%
PALMITOYLCARNITINE	10%
P-AMINOBENZOATE	67%
PANTOTHENATE	22%
PANTOTHENIC ACID	197%
P-CRESOL SULFATE	13%
PHENYLACETIC ACID	13%
PHENYLALANINE	5%
PHENYLGLUCOSIDE	13%
PHENYLLACTIC ACID	12%
PHENYLPYRUVATE	27%
PHOSHPATIDYLGLYCEROL	47%
PHOSPHATE	25%
PHOSPHOENOLPYRUVATE	69%
PHOSPHORYLCHOLINE	9%
PHOSPHOSERINE	57%
PHOSPHOTYROSINE	46%
P-HYDROXYBENZOATE	163%
PIMELYLCARNITINE	35%
PIPECOLIC ACID	3%
PREGNANOLONE SULFATE	12%
PREGNENOLONE SULFATE	17%
PROLINE	3%
PROLINE BETAINE	3%
PROPIONYLCARNITINE	3%

© 2024 Nagel EM et al. *JAMA Network Open.*

PROPIONYLCHOLINE	39%
PURINE	76%
PUTRESCINE	109%
PYRIDOXAMINE	70%
PYRIDOXINE	64%
PYROPHOSPHATE	166%
PYRUVATE	21%
QUINATE	42%
QUINOLINATE	31%
RHAMNOSE	48%
RIBITOL	45%
RIBOFLAVIN	15%
RIBOSYLIMIDAZOLEACETATE	18%
S1P	5%
S-2-HYDROXYETHYL-N-ACETYLCYSTEINYLCARNITINE	68%
S-ADENOSYL-L-METHIONINAMINE	61%
S-ADENOSYL-L-METHIONINE	47%
SALICYLURATE	31%
SARCOSINE	4%
SCYLLO-INOSITOL	118%
SERINE	7%
SHIKIMATE	69%
SHIKIMATE-3-PHOSPHATE	67%
S-Methyl propanethioate	74%
S-METHYL-5-THIOADENOSINE	36%
S-METHYLDEOXYCYTIDINE	184%
SN-GLYCEROL-3-PHOSPHATE	35%
SPERMIDINE	77%
SPERMINE	78%
SPHINGOSINE 1-PHOSPHATE	20%
S-RIBOSYL-L-HOMOCYSTEINE	55%
STEARAMIDE	35%

© 2024 Nagel EM et al. *JAMA Network Open.*

STEARATE	36%
STEAROYLCARNITINE	37%
SUBERIC ACID	47%
SUCCINATE	26%
SUCCINYLACETONE	69%
SUCCINYLADENOSINE	24%
SUCROSE	45%
SULFOLITHOCHOLYLGLYCINE	11%
SULFOPHENOL	13%
TARTARIC ACID	56%
TAURINE	3%
TAUROCHOLIC ACID	37%
TETRADECADIENCARNITINE	67%
TETRADECANEDIOATE	79%
TETRADECANOATE	42%
TETRADECENOYLCARNITINE	39%
THIAMINE	70%
THIAMINE-PHOSPHATE	67%
THREONINE	4%
THYMIDINE	65%
THYMINE	32%
TIGLYLCARNITINE	10%
TIGLYLGLYCINE	22%
TMAO	3%
TRANS-2-DODECENOYLCARNITINE	10%
TRANS-CINNAMIC ACID	14%
TRIDECANOYLCARNITINE	27%
TRIMETHYLLYSINE	9%
TRYPTOPHAN	5%
TYROSINE	5%
UDP-D-GLUCOSE	79%
UDP-D-GLUCURONATE	69%

© 2024 Nagel EM et al. *JAMA Network Open.*

UDP-N-ACETYL-GLUCOSAMINE	89%
UMP	91%
UNDECANEDIOIC ACID	20%
URACIL	22%
UREA	3%
UREIDOISOBUTYRIC ACID	11%
URIC ACID	5%
URIDINE	21%
VALINE	6%
XANTHINE	24%
XANTHOSINE	38%
XANTHURENIC ACID	65%

© 2024 Nagel EM et al. *JAMA Network Open.*

eTable 2. Non-GDM participant characteristics with *P* values (n=395)

	Included (n=242)	Not Included (n=153)	P-value
**Data**	**N (%) or Mean ± SD**		
Study site			**<0.001**
Minnesota	138 (57)	117 (76)	
Oklahoma	104 (43)	36 (24)	
Age, years	30.8 ± 3.9	31.0 ± 4.4	0.70
Pre-pregnancy BMI (kg/m^2)	26.8 ± 5.7	26.4 ± 5.2	0.46
BMI < 25 kg/m^2	98 (41)	75 (49)	0.28
BMI 25-29 kg/m^2	79 (33)	43 (28)	
BMI $\geq$ 30 kg/m^2	63 (26)	35 (23)	
Infant gestational age at birth (weeks)	39.7 ± 1.1	39.8 ± 1.2	0.63
Birth Anthropometrics			
Weight z-score	0.52 ± 0.86	0.36 ± 0.87	0.08
Length z-score	1.24 ± 1.20	0.97 ± 1.32	**0.04**
Weight for Length z-score	-0.69 ± 1.45	-0.64 ± 1.37	0.72
Infant sex			0.51
Male	130 (54)	77 (50)	
Female	112 (46)	76 (50)	

BMI, body mass index; GDM, gestational diabetes mellitus

Comparison between non-GDM participants (white) included in the present study and non-GDM participants not included in the present study.

© 2024 Nagel EM et al. *JAMA Network Open.*

eTable 3. Missing data from table 1

Variable	Number Missing
maternal age	4 (GDM: 1, non-GDM: 3)
maternal race	2 (GDM: 0, non-GDM: 2)
maternal ethnicity	2 (GDM: 0, non-GDM:2)
maternal education	12 (GDM: 0; non-GDM: 12)
parity	9 (GDM: 2, non-GDM: 7)
maternal pre-pregnancy BMI	2 (GDM:0, non-GDM: 2)
gestational weight gain	17 (GDM: 5, non-GDM: 12)
postpartum weight loss at 1 month	18 (GDM: 5, non-GDM: 13)
delivery category	6 (GDM: 0, non-GDM: 6)
exclusive breastfeeding at 3 months	21 (GDM: 8, non-GDM: 13)
exclusive breastfeeding at 6 months	34 (GDM: 12, non-GDM: 22)
Diet quality score	66 (GDM: 8; non-GDM: 58)
OGCT	58 (GDM: 23, non-GDM: 35)
gestational age	2 (GDM: 0, non-GDM: 2)
birth weight	4 (GDM: 0, non-GDM: 4)
birth length and birth weight for length	16 (GDM: 4, non-GDM: 12)
rapid weight gain	38 (GDM:12, non-GDM: 26)
sex	0
FFM, FM, %BF	16 (GDM: 4, non-GDM: 12)

© 2024 Nagel EM et al. *JAMA Network Open.*

eTable 4. Participant characteristics with *P* values (n=348)

	GDM (n=53)	Non-GDM (n=295)	P-value
**Data**	**N (%) or Mean ± SD**		
Study site			<.001
Minnesota	50 (94)	177 (60)	
Oklahoma	3 (6)	118 (40)	
Age, years	34.0 ± 4.3	30.7 ± 4.1	<.001
Race			0.06
White	37 (71)	242 (83)	
Other races[a]	15 (29)	48 (17)	
Ethnicity			1.0
Hispanic/Latino	2 (4)	9 (3)	
Not Hispanic/Latino	51 (96)	282 (96)	
Unknown	0	2 (1)	
Education			0.91
High school/GED/Associate's degree	12 (23)	72 (25)	
Bachelor's degree	21 (40)	110 (39)	
Graduate degree	20 (37)	101 (36)	
Annual income status			0.01
Less than $60,000	12 (23)	92 (33)	
$60,000-$90,000	6 (11)	69 (24)	
>$90,000	35 (66)	122 (43)	
Parity			1.0
0	19 (37)	109 (38)	
≥1	32 (63)	179 (62)	
Pre-pregnancy BMI (kg/m^2)[e]	28.8 (11.3)	26.0 (7.8)	0.01
BMI < 25 kg/m^2	18 (34)	118 (40)	
BMI 25-29 kg/m^2	10 (19)	95 (32)	
BMI $\geq$ 30 kg/m^2	25 (47)	80 (27)	
Oral Glucose Challenge Test (OGCT; mg/dL)	159.0 ± 19.8	106.7 ± 18.2	<.001
Gestational weight gain (kg)	9.52 ± 4.76	12.10 ± 6.75	0.01
Postpartum weight loss at 1 month (kg)	9.73 ± 2.57	8.54 ± 4.76	0.10
Mode of delivery			0.02
Vaginal	31 (58)	218 (75)	
Cesarean section	22 (42)	71 (25)	
Exclusive breastfeeding			
3 months	41 (91)	263 (93)	0.83
6 months	32 (78)	205 (75)	0.83
Diet quality score	62.5 ± 10.9	65.3 ± 8.8	0.10
Infant gestational age at birth (weeks)[e]	39.0 (1.3)	39.7 (1.4)	<.001
Birth Anthropometrics			
Weight (kg)	3.31 ± 0.42	3.53 ± 0.44	0.001
Weight z-score	0.016 ± 0.83	0.46 ± 0.88	0.001

© 2024 Nagel EM et al. *JAMA Network Open.*

Length (cm)	51.1 ± 2.3	51.7 ± 2.2	0.06
Length z-score	0.83 ± 1.19	1.15 ± 1.18	0.08
Weight for Length z-score	-0.91 ± 1.43	-0.68 ± 1.39	0.29
Rapid infant weight gain from 0-6 months[b]	4 (10)	39 (15)	0.56
Infant sex			0.68
Male	26 (49)	157 (53)	
Female	27 (51)	138 (47)	
Infant fat mass, kg			
1 month[c]	0.77 ± 0.32	0.80 ± 0.29	0.63
3 months[c]	1.48 ± 0.47	1.48 ± 0.41	0.99
6 months[d]	2.77 ± 0.68	2.77 ± 0.50	0.95
Infant fat-free mass, kg			
1 month[c]	3.54 ± 0.43	3.75 ± 0.44	0.002
3 months[c]	4.49 ± 0.49	4.69 ± 0.52	0.01
6 months[d]	5.47 ± 0.63	5.31 ± 0.59	0.11
Infant percent body fat			
1 month[c]	17.5 ± 5.3	17.2 ± 4.9	0.73
3 months[c]	24.5 ± 5.3	23.7 ± 4.8	0.33
6 months[d]	33.3 ± 4.9	34.0 ± 3.4	0.28

GDM, gestational diabetes mellitus

[a]Non-GDM: American Indian/Alaska Native 1%, Asian 3%, Black or African American 5%, Other 4%, More than one race 3%, Unknown 1%; GDM: American Indian/Alaska Native 2%, Asian 19%, Black or African American 4%, Other 2%, More than one race 2%, Unknown 2%
[b]Rapid weight gain: change in weight for age z-score from birth to 6 months > 0.67
[c]Obtained via air displacement plethysmography
[d]Obtained via DXA
[e]Median (IQR)

© 2024 Nagel EM et al. *JAMA Network Open.*

eTable 5. Unadjusted associations between maternal gestational diabetes status and 1-month human milk metabolites n=348

Metabolite	Class	Estimate[a] (AU)	SE	P-value	FDR
Glycine	Carboxylic acid and derivatives	-0.18	0.04	<.001	**0.002**
2-Hydroxybutyric acid	Hydroxy acids and derivatives	0.12	0.03	<.001	**0.004**
Stearoylcarnitine	Fatty acyls	-0.45	0.11	<.001	**0.004**
4-Oxodecanedioate	Fatty acyls	-0.17	0.05	0.001	0.05
Uric acid	Imidazopyrimidines	0.08	0.02	0.001	0.05
5-Hydroxyindoleacetic acid	Indoles and derivatives	-0.19	0.06	0.001	0.08
P-Aminobenzoate	Benzene and substituted derivatives	0.16	0.05	0.002	0.10
Asparagine	Carboxylic acid and derivatives	-0.16	0.05	0.002	0.11
Citramalate	Fatty acyls	0.31	0.10	0.002	0.11
O-Acetyl-L-serine	Carboxylic acid and derivatives	0.06	0.02	0.003	0.13
Thiamine	Organoheterocyclic compounds	-0.10	0.04	0.004	0.18
2-Octenoylcarnitine	Fatty acyls	-0.20	0.07	0.005	0.19
P-Hydroxybenzoate	Benzene and substituted derivatives	0.16	0.06	0.005	0.19
3-Carboxy-4-methyl-5-propyl-2-furanpropionate	Fatty acyls	0.13	0.05	0.006	0.19
8-Hydroxy-2-deoxyguanosine	Purine nucleosides	-0.08	0.03	0.006	0.19
Serine	Carboxylic acid and derivatives	-0.08	0.03	0.007	0.19
Leucine	Carboxylic acid and derivatives	0.10	0.04	0.007	0.19
Glutamic Acid	Carboxylic acids and derivatives	-0.12	0.04	0.008	0.20
Acetoacetate	Keto acids and derivatives	0.14	0.06	0.010	0.22
Hexanoic acid	Fatty acyls	0.12	0.05	0.010	0.22
Proline betaine	Carboxylic acid and derivatives	-0.28	0.11	0.009	0.22
Cystathionine	Carboxylic acid and derivatives	0.13	0.05	0.01	0.23
Furanedicarboxylcarnitine	Unknown	0.11	0.05	0.01	0.23
Lactate	Hydroxy acids and derivatives	0.10	0.04	0.01	0.23
Malate	Hydroxy acids and derivatives	0.08	0.03	0.01	0.23
Galactosylhydroxylysine	Carboxylic acid and derivatives	0.07	0.03	0.01	0.23
3-Hydroxyhexadecanoate	Fatty acyls	-0.07	0.03	0.02	0.26
4-Pyridoxic acid	Pyridines and derivatives	-0.12	0.05	0.02	0.26
Acetylcholine	Organonitrogen compounds	-0.05	0.02	0.02	0.26
Maleic acid	Dicarboxylic acids and derivatives	0.07	0.03	0.02	0.26
Methylmalonic acid	Dicarboxylic acids and derivatives	0.07	0.03	0.02	0.26
3-Methylphenylacetic acid	Benzene and substituted derivatives	0.12	0.05	0.02	0.26
4-Cresyl sulfate	Organic sulfuric acids and derivatives	-0.12	0.05	0.02	0.26
Deoxycholate	Steroids and steroid derivatives	-0.16	0.07	0.02	0.26
Fumarate	Dicarboxylic acids and derivatives	0.07	0.03	0.02	0.26

© 2024 Nagel EM et al. *JAMA Network Open.*

Tetradecanedioate	Fatty acyls	-0.13	0.06	0.02	0.26
Tiglylcarnitine	Fatty acyls	0.11	0.05	0.02	0.26
Glutamine	Carboxylic acid and derivatives	-0.10	0.05	0.03	0.29
Phenylacetic acid	Benzene and substituted derivatives	-0.08	0.04	0.03	0.29
Succinyladenosine	Purine nucleosides	0.04	0.02	0.02	0.29
FAPy-Adenine	Diazines	0.10	0.04	0.03	0.30
Pantothenate	Organooxygen compounds	0.08	0.03	0.03	0.30
3-Oxooctadecanoate	Fatty acyls	-0.12	0.05	0.03	0.30
Mevalonate-5-phosphate	Organic phosphoric acids and derivatives	0.09	0.04	0.03	0.30
2-Pyrrolidinone	Pyrrolidines	0.06	0.03	0.03	0.30
Phenylglucoside	Organooxygen compounds	0.06	0.03	0.03	0.30
1-Methyl-histidine	Carboxylic acids and derivatives	-0.07	0.04	0.03	0.32
2-Furoylglycine	Carboxylic acid and derivatives	-0.08	0.04	0.03	0.32
N4-Acetylcytidine	Pyrimidine nucleosides	-0.08	0.04	0.03	0.32
S1P	Sphingolipids	0.07	0.03	0.03	0.32
Propionylcarnitine	Fatty acyls	0.06	0.03	0.04	0.33
Guanidinosuccinic Acid	Carboxylic acid and derivatives	0.06	0.03	0.04	0.35
Hypusine	Carboxylic acid and derivatives	0.07	0.03	0.04	0.35
1,5-Anhydrosorbitol	Organooxygen compounds	-0.08	0.04	0.04	0.35
Ascorbic acid	Furanones	-0.43	0.21	0.04	0.35
D-Glyceraldehyde-3-phosphate	Organooxygen compounds	0.10	0.05	0.05	0.35
Glutamate	Carboxylic acid and derivatives	-0.04	0.02	0.05	0.35
Guanosine	Purine nucleosides	-0.06	0.03	0.04	0.35
N6-Acetyl-L-lysine	Carboxylic acid and derivatives	-0.07	0.04	0.05	0.35
N-Acetylglutamine	Carboxylic acid and derivatives	0.07	0.03	0.05	0.35
P-Cresol sulfate	Organic sulfuric acids and derivatives	-0.12	0.06	0.05	0.35
Pregnenolone sulfate	Steroids and steroid derivatives	-0.08	0.04	0.04	0.35
Tridecanoylcarnitine	Fatty acyls	-0.19	0.10	0.05	0.35
Cresol	Phenols	-0.10	0.05	0.05	0.35
D-Gluconate	Organooxygen compounds	-0.18	0.09	0.05	0.35
Hexadecanedioate	Fatty acyls	-0.10	0.05	0.05	0.35

AU, arbitrary units; GDM, gestational diabetes mellitus
[a]Estimates are for GDM vs non-GDM
Bold values indicate FDR <0.05

© 2024 Nagel EM et al. *JAMA Network Open.*

eTable 6. Adjusted associations between maternal GDM status and 1-month human milk metabolites n=322

Metabolite	Class	Estimate (AU)	SE	P-value	FDR
Stearoylcarnitine	Fatty acyls	-0.80	0.11	<.001	**<.001**
Phenylacetic acid	Benzene and substituted derivatives	-0.16	0.04	<.001	**0.005**
4-Cresyl sulfate	Organic sulfuric acids and derivatives	-0.20	0.05	<.001	**0.01**
Glycine	Carboxylic acid and derivatives	-0.16	0.04	<.001	**0.01**
P-Cresol sulfate	Organic sulfuric acids and derivatives	-0.24	0.07	<.001	**0.02**
Cresol	Phenols	-0.18	0.05	0.001	**0.04**
Pregnanolone sulfate	Steroids and derivatives	0.14	0.04	0.001	**0.04**
2-Hydroxybutyric acid	Hydroxy acids and derivatives	0.10	0.03	0.001	**0.048**
3-Methylphenylacetic acid	Benzene and substituted derivatives	0.19	0.06	0.001	**0.048**
8-Hydroxy-2-deoxyguanosine	Purine nucleosides	-0.10	0.03	0.002	0.06
9-Decenoylcarnitine	Fatty acyls	0.18	0.06	0.002	0.06
P-Aminobenzoate	Benzene and substituted derivatives	0.17	0.06	0.002	0.06
Proline betaine	Carboxylic acid and derivatives	-0.37	0.13	0.002	0.06
Uric acid	Imidazopyrimidines	0.08	0.04	0.002	0.06
Nonanedioate	Fatty acyls	0.16	0.06	0.004	0.12
Succinyladenosine	Purine nucleosides	0.06	0.02	0.004	0.12
Malate	Hydroxy acids and derivatives	0.09	0.03	0.006	0.16
4-Oxodecanedioate	Fatty acyls	-0.15	0.06	0.008	0.19
Leucine	Carboxylic acid and derivatives	0.11	0.04	0.008	0.19
Acetoacetate	Keto acids and derivatives	0.16	0.06	0.01	0.23
Citramalate	Fatty acyls	0.28	0.11	0.01	0.23
Hydroxyisocaproic acid	Fatty acyls	0.09	0.05	0.01	0.25
Maleic acid	Dicarboxylic acids and derivatives	0.08	0.03	0.01	0.25
2-Octenoylcarnitine	Fatty acyls	-0.20	0.08	0.01	0.25
2-Hydroxyvalerate	Fatty acyls	0.06	0.04	0.02	0.26
Deoxycholate	Steroids and steroid derivatives	-0.18	0.08	0.02	0.26
Fumarate	Dicarboxylic acids and derivatives	0.08	0.03	0.02	0.26
Furanedicarboxylcarnitine	Unknown	0.12	0.06	0.02	0.26
O-Acetyl-L-serine	Carboxylic acid and derivatives	0.05	0.02	0.02	0.26
3-Carboxy-4-methyl-5-propyl-2-furanpropionate	Fatty acyls	0.12	0.05	0.02	0.34

© 2024 Nagel EM et al. *JAMA Network Open.*

3-Hydroxyhexadecanoate	Fatty acyls	-0.08	0.03	0.03	0.34
Aconitate	Carboxylic acids and derivatives	0.09	0.04	0.02	0.34
Citrate	Carboxylic acids and derivatives	0.05	0.02	0.03	0.34
Cystathionine	Carboxylic acid and derivatives	0.12	0.05	0.03	0.34
FAPy-Adenine	Diazines	0.11	0.05	0.03	0.34
Glutamic Acid	Carboxylic acids and derivatives	-0.11	0.06	0.03	0.34
Methylmalonic acid	Dicarboxylic acids and derivatives	0.07	0.03	0.03	0.34
5-Hydroxyindoleacetic acid	Indoles and derivatives	-0.15	0.07	0.03	0.35
Dihydrouracil	Diazines	0.12	0.06	0.03	0.35
Mevalonate-5-phosphate	Organic phosphoric acids and derivatives	0.11	0.05	0.03	0.35
Ascorbic acid	Furanones	-0.49	0.23	0.04	0.36
ATP	Purine nucleotides	-0.36	0.17	0.04	0.36
D-Gluconate	Organooxygen compounds	-0.21	0.10	0.04	0.36
D-Sedoheptulose 7-phosphate	Organooxygen compounds	0.05	0.03	0.04	0.36
Guanosine	Purine nucleosides	-0.06	0.03	0.04	0.36
Lactose	Organooxygen compounds	0.03	0.02	0.04	0.36
N-Phenylacetyl glutamine	Carboxylic acids and derivatives	-0.09	0.04	0.04	0.36
Pantothenate	Organooxygen compounds	0.08	0.04	0.04	0.36
Putrescine	Organonitrogen compounds; amines	-0.12	0.06	0.04	0.36
Suberic acid	Fatty acyls	0.07	0.03	0.04	0.36
2-Pyrrolidinone	Pyrrolidines	0.06	0.04	0.05	0.39
3-Hydroxyoctanoylcarnitine	Fatty acyls	-0.13	0.06	0.05	0.39
D-Glucosamine 6-phosphate	Organooxygen compounds	-0.13	0.07	0.05	0.39
Guanidinosuccinic Acid	Carboxylic acid and derivatives	0.06	0.03	0.05	0.39
Palmitamide	Fatty acyls	0.09	0.04	0.05	0.39

AU, arbitrary units; GDM, gestational diabetes mellitus
Estimates are for GDM vs non-GDM
Bold values indicate FDR <0.05
Models adjusted for study center, maternal age, pre-pregnancy BMI, education, income, parity, and infant sex, and age at visit

© 2024 Nagel EM et al. *JAMA Network Open.*

eTable 7. Pathway Enrichment Analysis

Pathway Name	Match Status	P-value	FDR
Glyoxylate and dicarboxylate metabolism	5/11	0.002	0.15
Citrate cycle (TCA cycle)	4/9	0.01	0.21
Porphyrin metabolism	2/3	0.03	0.60
Valine, leucine and isoleucine degradation	3/8	0.04	0.60

Pathway analysis of milk metabolites significantly associated with maternal GDM by p<.05. Two metabolites (Furanedicarboxylcarnitine, 3-Hydroxyoctanoylcarnitine) not identified by MetaboAnalyst. We supplied our own reference metabolite, and 11 of 458 compounds were not identified by MetaboAnalyst based on KEGG ID.

© 2024 Nagel EM et al. *JAMA Network Open.*

eTable 8. Unadjusted associations between maternal OGCT results and 1-month human milk metabolites n=270

Metabolite	Class	Estimate[a] (AU)	SE	P-value	FDR
Asparagine	Carboxylic acid and derivatives	-0.004	0.001	<.001	**0.002**
Benzoate	Benzene and substituted derivatives	0.003	0.001	<.001	**0.003**
Indole-3-carboxylic acid	Indoles and derivatives	-0.003	0.001	<.001	0.06
Cyclic-AMP	Purine nucleotides	-0.004	0.001	0.001	0.09
Lactate	Hydroxy acids and derivatives	0.002	0.001	0.001	0.10
5-Hydroxyindoleacetic acid	Indoles and derivatives	-0.003	0.001	0.002	0.12
4-Oxodecanedioate	Fatty acyls	-0.002	0.001	0.003	0.15
O-Acetyl-L-serine	Carboxylic acid and derivatives	0.001	0.000	0.003	0.15
Deoxyguanosine	Purine nucleosides	0.003	0.001	0.004	0.16
Hydroxyphenylacetylglycine	Carboxylic acids and derivatives	0.003	0.001	0.004	0.16
Malonylcarnitine	Fatty acyls	0.002	0.001	0.004	0.15
P-Hydroxybenzoate	Benzene and substituted derivatives	0.003	0.001	0.003	0.16
Acetylcholine	Organonitrogen compounds	-0.001	0.000	0.005	0.16
Imidazolepropionic acid	Azoles	0.004	0.001	0.005	0.16
Palmitoylcarnitine	Fatty acyls	0.002	0.001	0.005	0.16
Stearoylcarnitine	Fatty acyls	-0.005	0.002	0.006	0.16
2-Hydroxybutyric acid	Hydroxy acids and derivatives	0.001	0.000	0.006	0.16
Glycine	Carboxylic acid and derivatives	-0.002	0.001	0.007	0.16
Phenylglucoside	Organooxygen compounds	0.001	0.000	0.007	0.16
S1P	Sphingolipids	0.001	0.001	0.007	0.16
3-Hydroxyhexadecanoate	Fatty acyls	-0.001	0.001	0.009	0.16
Anthranilate	Benzene and substituted derivatives	-0.002	0.001	0.008	0.16
L-Arginine	Carboxylic acid and derivatives	0.001	0.000	0.009	0.16
Cresol	Phenols	-0.002	0.001	0.009	0.16
Dodecanedioic acid	Fatty acyls	0.001	0.000	0.008	0.16
Maleic acid	Dicarboxylic acids and derivatives	0.001	0.000	0.009	0.16
3-Dehydroxycarnitine	Fatty acyls	-0.001	0.000	0.01	0.16
Hexanoic acid	Fatty acyls	0.002	0.001	0.01	0.17
NADPH	(5'->5')-dinucleotides	0.002	0.001	0.01	0.18
Fumarate	Dicarboxylic acids and derivatives	0.001	0.000	0.01	0.21
Guanidinosuccinic Acid	Carboxylic acid and derivatives	0.001	0.000	0.01	0.21
Threonine	Carboxylic acid and derivatives	-0.001	0.000	0.01	0.21
Histidinol	Organonitrogen compounds	-0.002	0.001	0.02	0.21
N-Acety-L-aspartic acid	Carboxylic acid and derivatives	0.001	0.001	0.02	0.21
Deoxycholate	Steroids and steroid derivatives	-0.003	0.001	0.02	0.21
Decanoylcarnitine	Fatty acyls	0.001	0.000	0.02	0.22

© 2024 Nagel EM et al. *JAMA Network Open.*

Metabolite	Class	Estimate[a]	SE	P-value	FDR
Uridine	Pyrimidine nucleosides	0.001	0.001	0.018	0.22
Methylimadazoleacetic acid	Azoles	-0.002	0.001	0.019	0.23
GAR	Glycinamide ribonucleotides	0.002	0.001	0.02	0.23
Serine	Carboxylic acid and derivatives	-0.001	0.000	0.02	0.23
2-Methylglutaric acid	Fatty acyls	-0.002	0.001	0.02	0.23
Riboflavin	Pteridines and derivatives	-0.004	0.002	0.02	0.23
Glutamine	Carboxylic acid and derivatives	-0.002	0.001	0.02	0.23
N-Acetylasparagine	Carboxylic acid and derivatives	0.002	0.001	0.02	0.25
4-Cresyl sulfate	Organic sulfuric acids and derivatives	-0.002	0.001	0.03	0.26
4-Pyridoxic acid	Pyridines and derivatives	-0.002	0.001	0.03	0.26
Bilirubin	Tetrapyrroles and derivatives	0.002	0.001	0.03	0.26
Deoxyinosine	Purine nucleosides	0.002	0.001	0.03	0.26
Furanedicarboxylcarnitine	Unknown	0.002	0.001	0.03	0.26
Galactosylhydroxylysine	Carboxylic acid and derivatives	0.001	0.000	0.03	0.26
Malate	Hydroxy acids and derivatives	0.001	0.000	0.03	0.26
Methylsuccinic acid	Fatty acyls	0.001	0.001	0.03	0.26
Propionylcholine	Organonitrogen compounds	-0.002	0.001	0.03	0.26
Salicylurate	Benzene and substituted derivatives	-0.002	0.001	0.03	0.26
Sulfophenol	Unknown	0.001	0.001	0.03	0.26
Beta-Guanidinopropionate	Guanidines	-0.001	0.000	0.03	0.27
Isobutyrl-L-carnitine	Fatty acyls	-0.002	0.001	0.04	0.28
Uric acid	Imidazopyrimidines	0.001	0.000	0.04	0.28
Methionine sulfoxide	Carboxylic acids and derivatives	-0.001	0.000	0.04	0.29
Thiamine	Organoheterocyclic compounds	-0.001	0.001	0.04	0.29
P-Aminobenzoate	Benzene and substituted derivatives	0.002	0.001	0.04	0.32
Dihydroorotate	Carboxylic acid and derivatives	-0.002	0.001	0.04	0.32
3-Oxooctadecanoate	Fatty acyls	-0.002	0.001	0.05	0.34

AU (arbitrary units); OGCT, oral glucose challenge test
Bold values indicate FDR <0.05
Beta estimates are showing the increase in AU (arbitrary units) of the metabolite concentration per mg/dl increase in OGCT

© 2024 Nagel EM et al. *JAMA Network Open.*

eTable 9. Adjusted associations between maternal OGCT results and 1-month human milk metabolites n=270

Metabolite	Class	Estimate[a] (AU)	SE	P-value	FDR
Stearoylcarnitine	Fatty acyls	-0.008	0.002	<.001	**<.001**
Nonanedioate	Fatty acyls	0.003	0.001	<.001	**0.01**
Indole-3-carboxylic acid	Indoles and derivatives	-0.003	0.001	<.001	**0.02**
Pregnanolone sulfate	Steroids and derivatives	0.002	0.001	<.001	**0.02**
Uridine	Pyrimidine nucleosides	0.002	0.001	<.001	**0.02**
Asparagine	Carboxylic acid and derivatives	-0.003	0.001	0.001	**0.049**
9-Decenoylcarnitine	Fatty acyls	0.003	0.001	0.001	0.07
Cresol	Phenols	-0.003	0.001	0.001	0.07
Salicylurate	Benzene and substituted derivatives	-0.002	0.001	0.002	0.11
L-Arginine	Carboxylic acid and derivatives	0.001	0.000	0.004	0.15
Cyclic-AMP	Purine nucleotides	-0.004	0.001	0.003	0.15
4-Cresyl sulfate	Organic sulfuric acids and derivatives	-0.002	0.001	0.007	0.18
4-Oxodecanedioate	Fatty acyls	-0.002	0.001	0.007	0.18
5-Hydroxyindoleacetic acid	Indoles and derivatives	-0.003	0.001	0.008	0.18
Benzoate	Benzene and substituted derivatives	0.002	0.001	0.006	0.18
Dodecanedioic acid	Fatty acyls	0.001	0.000	0.005	0.18
Hydroxyphenylacetylglycine	Carboxylic acids and derivatives	0.002	0.001	0.008	0.18
Lactate	Hydroxy acids and derivatives	0.002	0.001	0.005	0.18
Malonylcarnitine	Fatty acyls	0.002	0.001	0.008	0.18
O-Acetyl-L-serine	Carboxylic acid and derivatives	0.001	0.000	0.007	0.18
N-Acetylalanine	Carboxylic acid and derivatives	-0.002	0.001	0.009	0.20
Phenylacetic acid	Benzene and substituted derivatives	-0.002	0.001	0.01	0.21
Anthranilate	Benzene and substituted derivatives	-0.002	0.001	0.01	0.21
Decanoylcarnitine	Fatty acyls	0.001	0.000	0.01	0.21
Guanidinosuccinic Acid	Carboxylic acid and derivatives	0.001	0.001	0.01	0.21
NADPH	(5'->5')-dinucleotides	0.002	0.001	0.01	0.24
Phenylglucoside	Organooxygen compounds	0.001	0.000	0.02	0.26
Deoxycholate	Steroids and steroid derivatives	-0.003	0.001	0.02	0.28
3-Hydroxyhexadecanoate	Fatty acyls	-0.001	0.001	0.02	0.29
N-Acetylasparagine	Carboxylic acid and derivatives	0.002	0.001	0.02	0.29
Deoxyguanosine	Purine nucleosides	0.002	0.001	0.02	0.33
N-Acetylaspartic acid	Carboxylic acid and derivatives	0.001	0.001	0.02	0.33
Palmitoylcarnitine	Fatty acyls	0.002	0.001	0.02	0.33
Suberic acid	Fatty acyls	0.001	0.000	0.03	0.35
Hexanoic acid	Fatty acyls	0.002	0.001	0.03	0.38

© 2024 Nagel EM et al. *JAMA Network Open.*

Furanedicarboxylcarnitine	Unknown	0.002	0.001	0.03	0.40
Propionylcholine	Organonitrogen compounds	-0.002	0.001	0.03	0.40
Xanthine	Imidazopyrimidines	0.003	0.002	0.03	0.41
1H-Indole-3-carboxaldehyde	Organoheterocyclic compounds	-0.003	0.001	0.04	0.41
Deoxyadenosine	Purine nucleosides	-0.002	0.001	0.04	0.41
Galactosylhydroxylysine	Carboxylic acid and derivatives	0.001	0.000	0.04	0.41
Maleic acid	Dicarboxylic acids and derivaties	0.001	0.001	0.04	0.43
Dopamine	Phenols	0.001	0.000	0.04	0.44
Dihdroxyphenylalanine	Carboxylic acids and derivatives	0.001	0.000	0.05	0.46
Fructose 6-phosphate	Organooxygen compounds	0.002	0.001	0.05	0.46
GAR	Glycinamide ribonucleotides	0.002	0.001	0.05	0.46
Isobutyrl-L-carnitine	Fatty acyls	-0.002	0.001	0.05	0.46

AU (arbitrary units); OGCT, oral glucose challenge test

[a]Models adjusted for study center, maternal age, pre-pregnancy BMI, education, income, parity, and infant sex, and age at visit

Beta estimates are showing the increase in AU (arbitrary units) of the metabolite concentration per mg/dl increase in OGCT

© 2024 Nagel EM et al. *JAMA Network Open.*

eTable 10. Associations between milk metabolites and infant growth and body composition

	β estimates ± SE									
Metabolite	**ΔWLZ birth-6 mo[a]**	**P-value**	**ΔLAZ birth-6 mo[a]**	**P-value**	**Δ%BF 1-3 mo[b]**	**P-value**	**%BF 6 mo[c]**	**P-value**	**FFMI 6 mo[c]**	**P-value**
**2-Hydroxybutyric acid**										
Unadjusted	-0.21 ± 0.21	0.33	-0.03 ± 0.17	0.88	-1.30 ± 0.57	**0.03**	-0.59 ± 0.50	0.24	0.12 ± 0.13	0.35
Adjusted	0.003 ± 0.22	0.99	-0.26 ± 0.17	0.12	-1.50 ± 0.66	**0.03**	-0.32 ± 0.55	0.56	-0.01 ± 0.14	0.92
**3-Methylphenylacetic acid**										
Unadjusted	-0.02 ± 0.11	0.83	-0.08 ± 0.08	0.37	-0.16 ± 0.31	0.60	0.33 ± 0.25	0.20	-0.06 ± 0.07	0.38
Adjusted	-0.001 ± 0.11	0.99	-0.07 ± 0.09	0.44	-0.04 ± 0.34	0.90	0.42 ± 0.27	0.13	-0.02 ± 0.07	0.81
**4-Cresyl sulfate**										
Unadjusted	-0.06 ± 0.11	0.59	0.02 ± 0.09	0.82	0.41 ± 0.33	0.21	0.43 ± 0.28	0.12	0.02 ± 0.07	0.79
Adjusted	-0.21 ± 0.13	0.10	0.08 ± 0.10	0.41	0.39 ± 0.37	0.29	0.14 ± 0.31	0.66	0.03 ± 0.08	0.70
**Glycine**										
Unadjusted	-0.09 ± 0.15	0.56	0.09 ± 0.12	0.45	0.04 ± 0.41	0.93	0.72 ± 0.36	**0.04**	-0.04 ± 0.09	0.70
Adjusted	-0.13 ± 0.16	0.41	0.18 ± 0.12	0.13	-0.10 ± 0.46	0.82	0.31 ± 0.39	0.42	-0.005 ± 0.10	0.96
Cresol										
Unadjusted	-0.07 ± 0.12	0.57	0.08 ± 0.09	0.38	0.48 ± 0.34	0.16	0.45 ± 0.28	0.12	0.03 ± 0.07	0.69
Adjusted	-0.23 ± 0.13	0.07	0.14 ± 0.10	0.14	0.45 ± 0.38	0.24	0.22 ± 0.32	0.48	0.04 ± 0.08	0.63
**P-Cresol sulfate**										
Unadjusted	-0.10 ± 0.10	0.35	0.07 ± 0.08	0.37	0.09 ± 0.30	0.75	0.09 ± 0.24	0.72	0.02 ± 0.06	0.73
Adjusted	-0.24 ± 0.11	0.03[d]	0.13 ± 0.08	0.13	0.05 ±0.33	0.87	-0.18 ±0.28	0.51	0.002 ± 0.07	0.98
**Phenylacetic acid**										
Unadjusted	-0.23 ± 0.16	0.16	0.18 ± 0.13	0.15	0.35 ± 0.45	0.43	0.11 ±0.38	0.77	0.08 ± 0.10	0.41
Adjusted	-0.37 ± 0.17	0.04[d]	0.27 ± 0.13	**0.04**	0.32 ± 0.51	0.53	-0.23 ± 0.42	0.58	0.06 ± 0.10	0.59
**Pregnanolone sulfate**										
Unadjusted	0.002 ± 0.10	0.98	-0.08 ± 0.08	0.31	-0.19 ± 0.29	0.51	-0.16 ± 0.25	0.53	-0.10 ± 0.07	0.12
Adjusted	-0.12 ± 0.17	0.48	0.10 ± 0.13	0.44	0.60 ± 0.51	0.24	-0.80 ±0.42	0.06	0.005 ± 0.11	0.96
**Stearoylcarnitine**										
Unadjusted	0.13 ± 0.05	**0.01**	-0.01 ± 0.04	0.89	0.23 ± 0.15	0.13	0.12 ± 0.13	0.35	-0.01 ± 0.03	0.73
Adjusted	0.15 ± 0.06	0.02[d]	-0.01 ± 0.05	0.80	0.07 ± 0.19	0.71	0.03 ± 0.15	0.83	-0.05 ± 0.04	0.21

%BF, percent body fat; FFMI, fat-free mass index; GDM, gestational diabetes mellitus; LAZ, length for age z-score; WLZ, weight for length z-score

Bold values indicate significance at p<.05

[a]Adjusted for study center, parity, delivery category, income category, education category, maternal pre-pregnancy BMI, maternal age., and GDM status and infant gestational age at birth

[b]Adjusted for study center, parity, delivery category, income category, education category, maternal pre-pregnancy BMI, maternal age, GDM status, and infant age gestational age at birth, age at 3 month study visit, and sex

© 2024 Nagel EM et al. *JAMA Network Open.*

[c]Adjusted for study center, parity, delivery category, income category, education category, maternal pre-pregnancy BMI, maternal age, , GDM status, and infant gestational age at birth, age at 6 month study visit, and sex
[d]model not significant
Unadjusted models: ΔWLZ (n=300); ΔLAZ (n=300); Δ%BF (n=302); %BF at 6 months (n=299); FFMI (n=299)
Adjusted models: ΔWLZ (n=275); ΔLAZ (n=275); Δ%BF (n=273); %BF at 6 months (n=270); FFMI (n=273)

© 2024 Nagel EM et al. *JAMA Network Open.*

eTable 11. Associations between milk metabolites associated with GDM and rapid infant weight gain

Metabolite	Rapid weight gain birth-6 months[a]
	Odds Ratio (95% CL)
**2-Hydroxybutyric acid**	
Unadjusted	0.84 (0.39, 1.81)
Adjusted	1.03 (0.42, 2.56)
**3-Methylphenylacetic acid**	
Unadjusted	0.95 (0.64, 1.41)
Adjusted	0.97 (0.61, 1.52)
**4-Cresyl sulfate**	
Unadjusted	1.36 (0.85, 2.16)
Adjusted	1.48 (0.85,2.58)
**Glycine**	
Unadjusted	0.98 (0.57, 1.68)
Adjusted	0.77 (0.41, 1.45)
Cresol	
Unadjusted	1.53 (0.94, 2.48)
Adjusted	1.73 (0.98, 3.06)
**P-Cresol sulfate**	
Unadjusted	1.14 (0.78, 1.67)
Adjusted	1.18 (0.75, 1.86)
**Phenylacetic acid**	
Unadjusted	1.25 (0.69, 2.27)
Adjusted	1.45 (0.72, 2.92)
**Pregnanolone sulfate**	
Unadjusted	1.30 (0.91, 1.86)
Adjusted	1.35 (0.69, 2.64)
**Stearoylcarnitine**	
Unadjusted	**1.35 (1.10, 1,65)**
Adjusted	**1.66 (1.23, 2.25)**

GDM, gestational diabetes mellitus

[a]Adjusted for study center, parity, delivery category, income category, education category, maternal pre-pregnancy BMI, breastfeeding exclusivity at 6 months postpartum, GDM status and infant gestational age at birth, age at 1 month visit, WAZ at birth, and sex
Unadjusted models (n = 310); adjusted models (n = 286)
Odds of rapid infant weight gain per increase of one arbitrary unit of each metabolite

© 2024 Nagel EM et al. *JAMA Network Open.*

eTable 12. Associations between OGCT and infant growth and body composition

**OGCT (mg/dL)**		
	**ΔWFLZ (birth-6 mo)[a]**	
	β estimates ± SE	P-value
Unadjusted	-0.003 ± 0.004	0.42
Adjusted	-0.004 ± 0.004	0.34
	**ΔLAZ (birth-6 mo)[a]**	
	β estimates ± SE	P-value
Unadjusted	0.0005 ± 0.003	0.89
Adjusted	0.001 ± 0.003	0.73
	**Δ%BF (1-3 mo)[b]**	
	β estimates ± SE	P-value
Unadjusted	0.006 ± 0.012	0.63
Adjusted	0.006 ± 0.013	0.63
	**%BF (6 months)[c]**	
	β estimates ± SE	P-value
Unadjusted	-0.002 ± 0.010	0.83
Adjusted	-0.003 ± 0.01	0.79
	**FFMI (6 months)[c]**	
	β estimates ± SE	P-value
Unadjusted	-0.002 ±0.003	0.45
Adjusted	-0.003 ± 0.003	0.33
	**RWG (0-6 months)[d]**	
	Odds Ratio (95% CL)	
Unadjusted	1.00 (0.99, 1.02)	
Adjusted	1.00 (0.98, 1.02)	

%BF, percent body fat; FFMI, fat-free mass index; GDM, gestational diabetes mellitus; LAZ, length for age z-score, OGCT, oral glucose control test, WFLZ, weight for length z-score
The units for beta estimates are increases in one unit of the dependent variable per mg/dl increase in OGCT.

[a]Adjusted for study center, parity, income category, education category, maternal pre-pregnancy BMI, and maternal age
[b]Adjusted for study center, parity, income category, education category, maternal pre-pregnancy BMI, maternal age, infant age at 3 month study visit, and sex
[c]Adjusted for study center, parity, income category, education category, maternal pre-pregnancy BMI, maternal age, age at 6 month study visit, and sex
[d]Adjusted for study center, parity, income category, education category, maternal pre-pregnancy BMI, and maternal age

Adjusted models: ΔWFLz (n=240); ΔLAZ (n=240); Δ%BF (n=239); %BF at 6 months (n=251); FFMI (n=236); Rapid Weight Gain (n=246)

© 2024 Nagel EM et al. *JAMA Network Open.*

eTable 13. Associations between GDM and infant body composition and growth variables

GDM		
	**ΔWFLZ (birth-6 mo)[a]**	
	β estimates ± SE	P-value
Unadjusted	-0.36 ± 0.27	0.19
Adjusted	-0.39 ± 0.28	0.16
	**ΔLAZ (birth-6 mo)[a]**	
	β estimates ± SE	P-value
Unadjusted	0.48 ± 0.21	**0.02**
Adjusted	0.48 ± 0.22	**0.03**
	**Δ%BF (1-3 mo)[b,e]**	
	β estimates ± SE	P-value
Unadjusted	0.15 ± 0.73	0.84
Adjusted	0.31 ± 0.80	0.70
	**%BF (6 months)[c,f]**	
	β estimates ± SE	P-value
Unadjusted	-0.68 ± 0.62	0.28
Adjusted	-0.84 ± 0.67	0.21
	**FFMI (6 months)[c]**	
Unadjusted	0.17 ± 0.16	0.27
Adjusted	-0.04 ± 0.17	0.81
	**RWG (0-6 months)[d]**	
	Odds Ratio (95% CL)	
Unadjusted	1.57 (0.53, 4.65)	
Adjusted	0.53 (0.14, 1.96)	

%BF, percent body fat; FFMI, fat-free mass index; GDM, gestational diabetes mellitus; LAZ, length for age z-score; RWG, rapid weight gain; WLZ, weight for length z-score

[a]Adjusted for study center, parity, income category, education category, maternal pre-pregnancy BMI, and maternal age
[b]Adjusted for study center, parity, income category, education category, maternal pre-pregnancy BMI, maternal age, infant age at 3 month study visit, and sex
[c]Adjusted for study center, parity, income category, education category, maternal pre-pregnancy BMI, maternal age, age at 6 month study visit, and sex
[d]Adjusted for study center, parity, income category, education category, maternal pre-pregnancy BMI, and maternal age
[e]Body composition via air displacement plethysmography
[f]Body composition at 6 months via dual energy x-ray absorptiometry

Rapid Weight Gain = ΔWAZ from 0-6 months > 0.67

Adjusted models: ΔWLZ (n=277); ΔLAZ (n=277); Δ%BF (n=280); %BF at 6 months (n=276); FFMI (n=276); RWG (n=286)

© 2024 Nagel EM et al. *JAMA Network Open.*
