# Supplementary material for: Gestational Diabetes, the Human Milk Metabolome, and Infant Growth and Adiposity
Source: JAMA Netw Open. 2024 Dec 12;7(12):e2450467. doi: 10.1001/jamanetworkopen.2024.50467 (PMC11638796; doi:10.1001/jamanetworkopen.2024.50467)
Supplement: Supplement 2. — Data Sharing Statement [file jamanetwopen-e2450467-s002.pdf]

Data Sharing Statement

Nagel. Gestational Diabetes, the Human Milk Metabolome, and Infant Growth and Adiposity. *JAMA Netw Open*. Published December 12, 2024. doi:10.1001/jamanetworkopen.2024.50467

Data
Data available: Yes
Data types: Deidentified participant data, Data dictionary
How to access data: Data will be available upon request to corresponding author (nagel127@umn.edu)
When available: With publication

Supporting Documents
Document types: None

Additional Information
Who can access the data: Data will be made available upon reasonable request
Types of analyses: For research purposes
Mechanisms of data availability: After approval by investigators
